# Differential contribution of bone marrow-derived infiltrating monocytes and resident macrophages to persistent lung inflammation in chronic air pollution exposure

**DOI:** 10.1038/s41598-020-71144-1

**Published:** 2020-09-01

**Authors:** Roopesh Singh Gangwar, Vinesh Vinayachandran, Palanivel Rengasamy, Ricky Chan, Bongsoo Park, Rachel Diamond-Zaluski, Elaine Ann Cara, Anthony Cha, Lopa Das, Courteney Asase, Andrei Maiseyeu, Jeffrey Deiuliis, Jixin Zhong, Wayne Mitzner, Shyam Biswal, Sanjay Rajagopalan

**Affiliations:** 1 Case Cardiovascular Research Institute, Case Western Reserve University, Cleveland, OH 44106 USA; 2 Institute for Computational Biology, Case Western Reserve University, Cleveland, OH 44106 USA; 3 Department of Environmental Health and Engineering, Johns Hopkins University School of Public Health, Baltimore, MD 21205 USA; 4 Division of Cardiovascular Medicine, University Hospitals, Harrington Heart and Vascular Institute (HHVI), Cleveland, OH USA; 5 Herman K. Hellerstein MD, Professor of Cardiovascular Research Department of Internal Medicine and Radiology, Case Cardiovascular Research Institute, Case Western Reserve University, 11100 Euclid Ave., Mailstop: 5038, Cleveland, OH 44106 USA

**Keywords:** Inflammation, Alveolar macrophages, Immunology, Environmental impact

## Abstract

Chronic exposure to particulate matter < 2.5µ (PM_2.5_) has been linked to cardiopulmonary disease. Tissue-resident (TR) alveolar macrophages (A*Φ*) are long-lived, self-renew and critical to the health impact of inhalational insults. There is an inadequate understanding of the impact of PM_2.5_ exposure on the nature/time course of transcriptional responses, self-renewal of A*Φ*, and the contribution from bone marrow (BM) to this population. Accordingly, we exposed chimeric (CD45.2/CD45.1) mice to concentrated PM_2.5_ or filtered air (FA) to evaluate the impact on these end-points. PM_2.5_ exposure for 4-weeks induced an influx of BM-derived monocytes into the lungs with no contribution to the overall TR-A*Φ* pool. Chronic (32-weeks) PM_2.5_ exposure on the other hand while associated with increased recruitment of BM-derived monocytes and their incorporation into the A*Φ* population, resulted in enhanced apoptosis and decreased proliferation of TR-A*Φ*. RNA-seq analysis of isolated TR-A*Φ* and BM-A*Φ* from 4- and 32-weeks exposed mice revealed a unique time-dependent pattern of differentially expressed genes. PM_2.5_ exposure resulted in altered histological changes in the lungs, a reduced alveolar fraction which corresponded to protracted lung inflammation. Our findings suggest a time-dependent entrainment of BM-derived monocytes into the A*Φ* population of PM_2.5_ exposed mice, that together with enhanced apoptosis of TR-A*Φ* and reorganization of transcriptional responses, could collectively contribute to the perpetuation of chronic inflammation.

## Introduction

Exposure to ambient air pollution, specifically particulate matter < 2.5 µm in diameter (PM_2.5_) is the world’s leading environmental risk factor for non-communicable diseases, including respiratory disorders. Inhalational exposure to PM_2.5_ has been implicated in adverse health outcomes across the lifespan, including impaired lung development, acceleration of age-related decline in lung function, pulmonary and cardiovascular disorders^1^. The alveolar macrophage (A*Φ*) population in the lung are the first line of defense, responsible for the phagocytosis of inhaled particles and maintenance of immune homeostasis in the lung. A*Φ* in mice, derived from the yolk sac and fetal liver monocytes, have a unique surface marker phenotype. They populate the alveolar space soon after birth and eventually express high levels of Siglec-F (Siglec-F^high^CD11c^high^) in mice^2,3^. In the murine lung micro-environment apart from A*Φ*, there are also alveolar myeloid dendritic cells (cDC), interstitial macrophages, and a small portion of residential lung monocytes.

The inflammatory response to injury and subsequent resolution of inflammation is tissue and context-dependent and orchestrated by monocytes and macrophages. The recruitment of bone marrow-derived (BM) monocytes into the site of inflammation and subsequent differentiation into macrophages is consistent with a stereotypical response to initial “injury”^4^. Under homeostatic conditions, A*Φ* of the lung are long-lived and self-renew to maintain their population without contribution from circulating adult bone marrow-derived monocytes^5,6^. However, in response to stimuli such as bleomycin-induced lung injury, it has been demonstrated that recruited BM-monocytes can differentiate into macrophages in the lung, and acquire the TR-A*Φ* phenotype^4^. Our group has previously reported that PM_2.5_ exposure promotes a monocyte egress from bone marrow and pro-inflammatory responses in lung and bronchoalveolar lavage fluid^7,8^. However, the relative contributions of recruited monocytes, their differentiation (if any) into A*Φ*, the fate of TR-A*Φ* (including their proliferation and self-renewal capacity) and finally their transcriptomic profile in response to PM_2.5_ exposure has not been previously described.

In this work we describe and characterize the inflammatory response to real-world chronic inhalational PM_2.5_ exposure, using a model of “lung shielded chimeric mice”, to distinguish TR-A*Φ* from BM-A*Φ* and attempt to delineate the unique transcriptomic signature of PM_2.5_ exposure in the A*Φ* population. We posit that a better understanding of the lung immune response with chronic inhalation to air pollution across the life span may provide much-needed insights into the chronic biology of an omnipresent risk factor.

## Results

### Short-term (4-week) PM_2.5_ exposure induces recruitment of bone marrow-derived monocytes into lung without evidence of differentiation into alveolar macrophages

Chimeric mice were generated by irradiating chest shielded C57BL/6J (CD45.2) mice and transplanted with bone marrow from C57BL/6J (CD45.1) mice (see Methods and Supplementary Fig. 1 for details). Chest shielding preserved the lungs from irradiation and thereby allowing all tissue-resident A*Φ* and myeloid cells in the lungs to remain as CD45.2 origin, while peripheral blood myeloid cells were replaced by CD45.1 bone marrow. Thus, chimeric mice allowed the ability to identify recruited cells of CD45.1 origin (from peripheral blood) into the lungs (CD45.2) (Supplementary Fig. 1b). For the ease of understanding and better readability, we will refer A*Φ* of CD45.2 origin as tissue-resident A*Φ* (TR-A*Φ*) and A*Φ* of CD45.1 origin as bone marrow-derived A*Φ* (BM-A*Φ*) throughout the manuscript.

**Figure 1:**
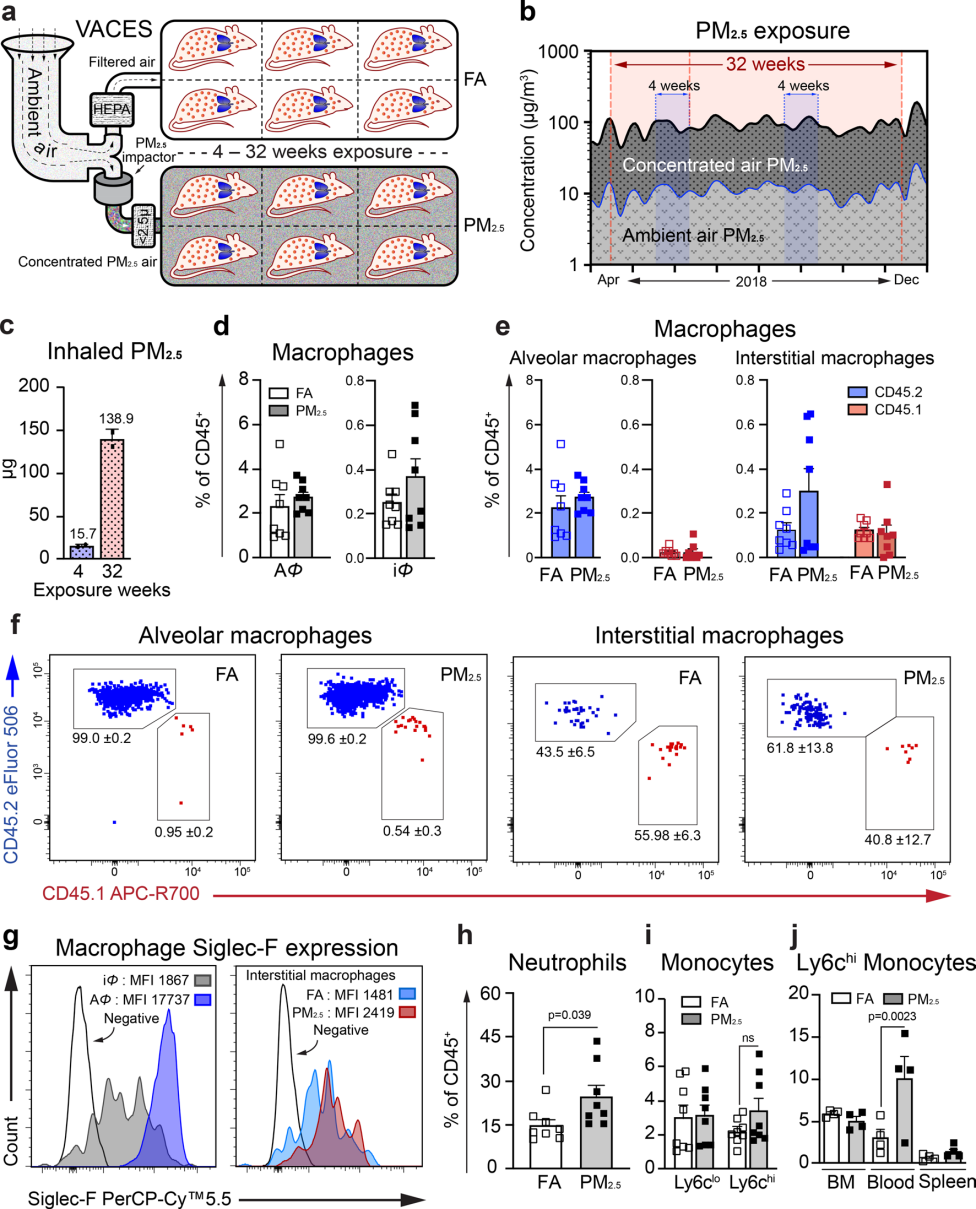
4-week PM_2.5_ exposure induces recruitment of bone marrow-derived monocytes into the lung without evidence of differentiation into alveolar macrophages. (**a**) Schematic representation of Versatile Aerosol Concentrator and Enrichment System (VACES) in which mice were exposed to filtered air (FA) or concentrated PM_2.5_ air (PM) for 4–32 weeks. (**b**) PM_2.5_ concentration in ambient air and inside VACES chambers during 4-weeks (blue shaded area) and 32-weeks (red shaded area) exposure time. (**c**) The total inhaled dose of PM_2.5_ during exposures. (**d**) Lung macrophage population (A*Φ* and i*Φ*). (**e**) A*Φ* and i*Φ* of tissue-resident (CD45.2, blue) and bone marrow (CD45.1, red) origin. (see also Supplementary Fig. 2) (**f**) Representative flow cytometry plots showing A*Φ* and i*Φ* of tissue-resident (blue) and BM-origin (red) showing frequencies of the respective population. (**g**) Expression of Siglec F, on i*Φ* and A*Φ* from FA (representative plot, left panel) and i*Φ* from FA and PM_2.5_ exposed mice (right panel). (**h**) Neutrophil population in the lungs. (**i**) Monocyte subsets (Ly6c^lo^ and Ly6c^hi^) in the lung. (**j**) Ly6c^hi^ monocytes populations from bone marrow, blood, and spleen. Data are represented as % of total CD45^+^ cells, mean ± SEM, from two independent experiments with 4–6 mice in each experiment. Data were analyzed with GraphPad prism v8.3 using Student’s *‘t’* test or one-way ANOVA with Bonferroni’s posthoc test for multiple comparisons, statistically significant *p* values (< 0.05) are mentioned for respective comparison.

Chimeric mice were exposed to filtered air (FA) or concentrated PM_2.5_ air (PM_2.5_) in VACES chambers for 4-weeks under controlled temperature and humidity, (Fig. 1a and Supplementary Fig. 1c) as described previously^8^. The mean daily PM_2.5_ concentration inside the chambers during exposure was 88.8 ± 14.5 μg/m^3^, while the daily ambient mean PM_2.5_ concentration was 10.8 ± 1.4 μg/m^3^ (Fig. 1b). This translates approximately into a total inhaled dose of PM_2.5_/mouse (4-weeks) of 15.66 ± 1.63 μg (given mice exposure of 6–8 h/day, 5 days/week, assuming a ventilatory rate of 105 breaths/min and average tidal volume is 0.2 cc/breath) (Fig. 1c). Post-exposure, macrophage (A*Φ* and interstitial macrophage), neutrophil, and monocyte population in the lungs, and monocytes in the blood, spleen, and BM were analyzed using multicolor flow cytometry (Supplementary Fig. 4). In the lungs of PM_2.5_ exposed chimeric mice, there was a mild increase in total lung macrophage population (alveolar and interstitial *Φ*) in response to PM_2.5_ exposure (Fig. 1d and Supplementary Fig. 2d). A*Φ*s in both PM_2.5_ and FA exposed mice were primarily (> 98%) of tissue-resident origin and a negligible amount of BM-origin with no difference in response to PM_2.5_ exposure for 4-weeks. Although there were interstitial macrophages (i*Φ*) of CD45.1 origin detected in the lungs, however, this population was comparable to CD45.2 origin i*Φ* in FA and PM_2.5_ exposed groups (Fig. 1e, f). The i*Φ* of PM_2.5_ exposed mice expressed higher levels of Siglec F (a characteristic marker of A*Φ*) compared to FA group at 4-weeks, suggesting the maturation of i*Φ* population towards A*Φ* (Fig. 1g). As an indication of acute inflammation higher neutrophil infiltration (*p* < 0.05) was noted in PM_2.5_ compared to FA exposed mice (Fig. 1h). Additionally, a non-significant increase in Ly6c^hi^ proinflammatory monocytes percentage and total monocyte percentages were detected in PM_2.5_ exposed mice (Fig. 1i and Supplementary Fig. 2c). There were no differences in total leukocytes (CD45^+^) in the lungs (Supplementary Fig. 2a). Percentage difference of the monocyte’s subsets in the periphery (blood and spleen) demonstrated significantly increased (*p* < 0.05) Ly6c^hi^ monocytes (%) in the blood in PM_2.5_ vs FA exposed mice (Fig. 1j).

### Chronic PM_2.5_ exposure (32-weeks) perturbs lung cellular immune homeostasis

**Figure 2:**
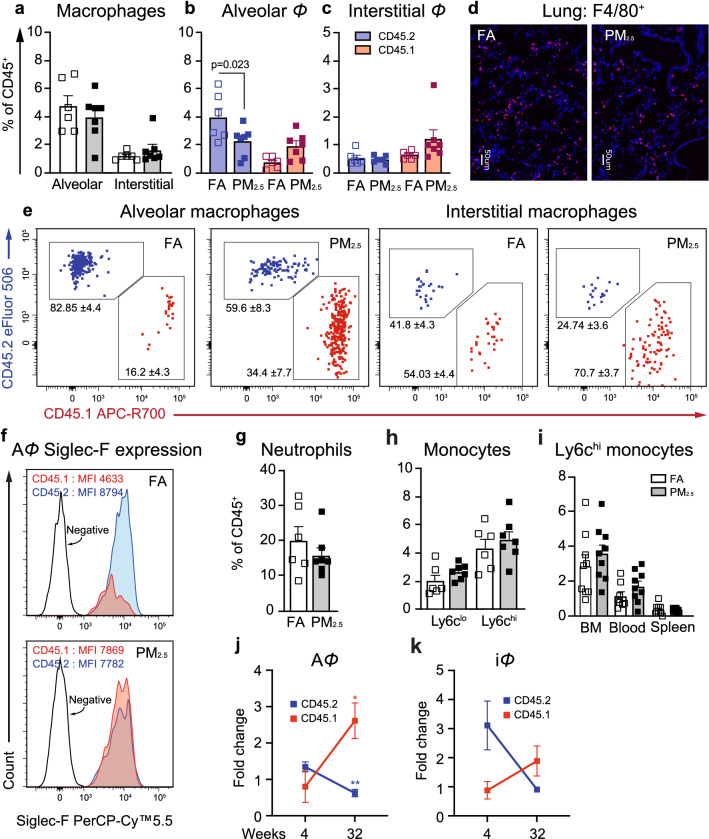
Chronic PM_2.5_ exposure (32-weeks) impairs lung cellular immune homeostasis. (**a**) Lung macrophage (A*Φ* and i*Φ*) population. (**b**) A*Φ* of tissue-resident (CD45.2, blue) and bone marrow (CD45.1, red) origin. (**c**) i*Φ* of tissue-resident and bone marrow origin. (see also Supplementary Fig. 3). (**d**) Lung tissue stained for F4/80, showing total *Φ* in representative sections from 32-weeks FA and PM_2.5_ exposed mice, scale bar 50 μm. (**e**) Representative flow cytometry plots showing A*Φ* and i*Φ* of tissue-resident (blue) and BM-origin (red) showing frequencies of the respective population. (**f**) Siglec F expression on TR-A*Φ* and BM-A*Φ*, representative plots from FA (left), and PM_2.5_ (right) exposed mice. (**g**) Neutrophil population in the lungs. **h.** Monocytes subsets (Ly6c^lo^ and Ly6c^hi^) in the lungs. (**i**) Ly6c^hi^ monocyte population from bone marrow, blood, and spleens. (**j**, **k**) Fold change in tissue-resident and BM-derived, (**j**) A*Φ* and (**i**) i*Φ* at 32-weeks over 4-weeks of PM_2.5_ exposure. Data are represented as % of total CD45^+^ cells, mean ± SEM, from two independent experiments with 4–6 mice in each experiment (**a**–**c**, **g**–**i**) and as fold change over control FA exposed mice (**j**, **k**). Data were analyzed with GraphPad prism v8.3 using Student’s *‘t’* test or one-way ANOVA with Bonferroni’s posthoc test for multiple comparisons, statistically significant *p* values (< 0.05) are mentioned for respective comparison.

To determine the effect of chronic PM_2.5_ exposure on TR-A*Φ* and recruitment of BM-A*Φ*, we exposed chimeric mice for 32-weeks in the VACES chambers (mean daily PM_2.5_ concentration in chambers was 90.1 ± 24.2 μg/m^3^ and ambient PM_2.5_ concentration was 11.5 ± 3.6 μg/m^3^, total inhaled PM_2.5_ dose was 138.89 ± 11.79 μg) (Fig. 1b, c). Total leukocytes (CD45^+^) and cell viability in the lungs of PM_2.5_ exposed mice were similar to FA exposed mice for 32-weeks (Supplementary Fig. 3a, b). Although the total macrophage population (A*Φ* and i*Φ*) in lungs, at 32-weeks of exposure remained the same in both the FA and PM_2.5_ groups (Fig. 2a, d and Supplementary Fig. 3d), there was a significant decrease (*p* < 0.05) in TR-A*Φ* % with a non-significant increase in BM-A*Φ*, and BM-i*Φ* % in PM_2.5_ mice compared to FA mice (Fig. 2b, c, e). BM-A*Φ* are reported to acquire the surface marker phenotype of TR-A*Φ*^4^ and we found that Siglec F expression on BM-A*Φ* in PM_2.5_ mice was similar to levels seen in TR-A*Φ* (Fig. 2f). There was no difference in the neutrophil population (Fig. 2g). Even though total monocytes and Ly6c^hi^ subsets in lungs were higher at 32-weeks compared to 4-weeks exposure, there was no differential increase in Ly6c^hi^ subset at 32-weeks in both the FA and PM_2.5_ exposed mice (Fig. 2h and Supplementary Fig. 3c), and correspondingly there was no difference in the Ly6c^hi^ monocytes in the blood, spleen, and BM in FA and PM_2.5_ exposed mice (Fig. 2i). Compared to 4-weeks exposure, BM-A*Φ* frequencies in 32-weeks PM_2.5_ exposed mice were increased by 3.3-fold (*p* < 0.05) with a reciprocal decrease in TR-A*Φ* by 0.46-fold (*p* < 0.01) and a similar trend was observed in i*Φ*, (Fig. 2j, k). Together, this data suggests that PM_2.5_ exposure results in the partial replacement of TR-A*Φ* by BM-A*Φ* in 32-weeks PM_2.5_ exposure.

#### Chronic PM_2.5_ exposure impairs alveolar macrophage self-renewal potential

**Figure 3:**
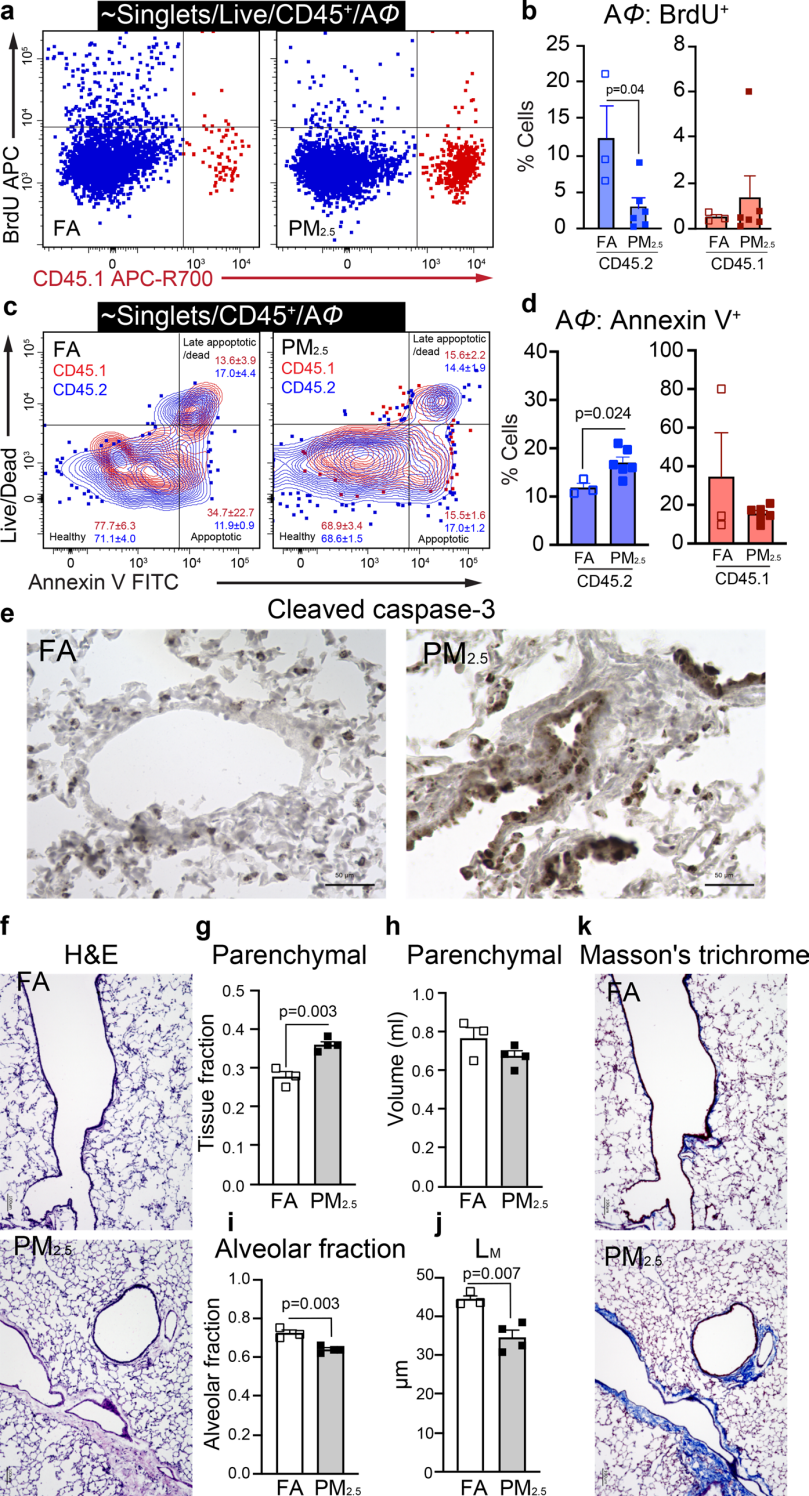
Chronic PM_2.5_ exposure impairs the proliferation potential and increased apoptosis in TR-A*Φ*. (**a**) Representative gating plots for BrdU^+^ in A*Φ* from 32-weeks of PM_2.5_ and FA exposed mice. (**b**) BrdU^+^ (proliferating) TR-A*Φ* and BM-A*Φ*. (**c**) Representative gating plots for Annexin V^+^ A*Φ* from 32-weeks of PM_2.5_ and FA exposed mice, showing frequencies of the respective population. (**d**) Annexin V^+^ (apoptotic) TR-A*Φ* and BM-A*Φ*. (**e**) Representative images of lung sections stained for Cleaved caspase-3 (apoptosis), scale bar 50 μm. (**f**) Representative images of lung sections stained for H&E (scale bar 100 μm) for lung morphometry quantitative analysis, (**g**–**j**) (see also Supplementary Fig. 5c). (**g**) Parenchymal tissue fraction, (**h**) Parenchymal volume. (**i**) The alveolar fraction of lung parenchyma. (**j**) Mean linear intercept of parenchymal airspaces (L_M_). (**k**) Representative images of lung sections stained for Masson’s trichrome (Fibrosis), scale bar 100 μm. Separate mice were used for histological and morphometric analysis, and BrdU and Annexin V staining. Data were analyzed with GraphPad prism v8.3 using Student’s *‘t’* test or one-way ANOVA with Bonferroni’s posthoc test for multiple comparisons, statistically significant *p* values (< 0.05) are mentioned for respective comparison.

Given the decrease in TR-A*Φ* in PM_2.5_ exposed mice at 32-weeks, we sought to elucidate the self-renewal capacity and maintenance of this cell population. In general since A*Φ* in the lungs are maintained by self-renewal^6^, we investigated the proliferative potential of A*Φ* by BrdU incorporation and apoptosis (Annexin V^+^) in a separate group of 32-weeks of PM_2.5_ and FA exposed mice. We specifically labeled the A*Φ* population by delivering the BrdU directly into the mouse lungs and quantified BrdU^+^ A*Φ*. PM_2.5_ mice showed a marked decrease in the BrdU^+^ TR-A*Φ* population as compared to FA mice, indicating an impaired proliferation of TR-A*Φ* (Fig. 3a, b). Interestingly, BrdU^+^ BM-A*Φs* were negligible, with no difference (in proliferation) between FA or PM_2.5_ exposed mice (Fig. 3a, b, and Supplementary Fig. 5a). Apoptosis in the A*Φ* population was examined using flow cytometry (Annexin V staining) and cleaved caspase-3 staining in lung histological sections. Increased Annexin V^+^ events (cells) in the TR-A*Φ* population were observed in PM_2.5_ compared to FA exposed mice indicating enhanced apoptosis of TR-A*Φ* with chronic PM_2.5_ exposure (Fig. 3c–e). Tissue histology (Cleaved caspase 3 staining) also showed overall increased apoptotic cells in PM_2.5_ *vs* FA mice (Fig. 3e). These observations indicate that chronic PM_2.5_ exposure impairs the self-renewal potential and increases the apoptosis of TR-A*Φ*.

#### PM_2.5_ exposure induces histological changes and reduces lung function

The primary lung function i.e. the gas exchange is critically dependent on the availability of the surface area of the interface establishing contact between the air in the alveoli and the blood in the alveolar capillaries. This can be quantified using physiological equations for predicting oxygen uptake morphometry analysis^9^. Lung sections were stained with H&E (Fig. 3f) and morphometric quantitation was done using STEPanizer v1.0 as described^10,11^. Parenchymal tissue fraction significantly increased (*p* < 0.01), with no differences in lung parenchymal volume in PM_2.5_ compared to FA exposed mice (Fig. 3g, h). The alveolar fraction was significantly reduced (*p* < 0.01) and mean linear intercept ‘L_M_’, a measure of gas exchange surface, was also significantly reduced by (*p* < 0.05) in PM_2.5_ exposed mice compared to FA mice (Fig. 3i, j). Masson’s trichrome staining revealed evidence of increased collagen surrounding the airways in the lung tissue in the PM_2.5_ exposed mice compared to FA exposed mice (Fig. 3k).

### Transcriptomic landscape of A*Φ* with PM_2.5_ exposure

**Figure 4:**
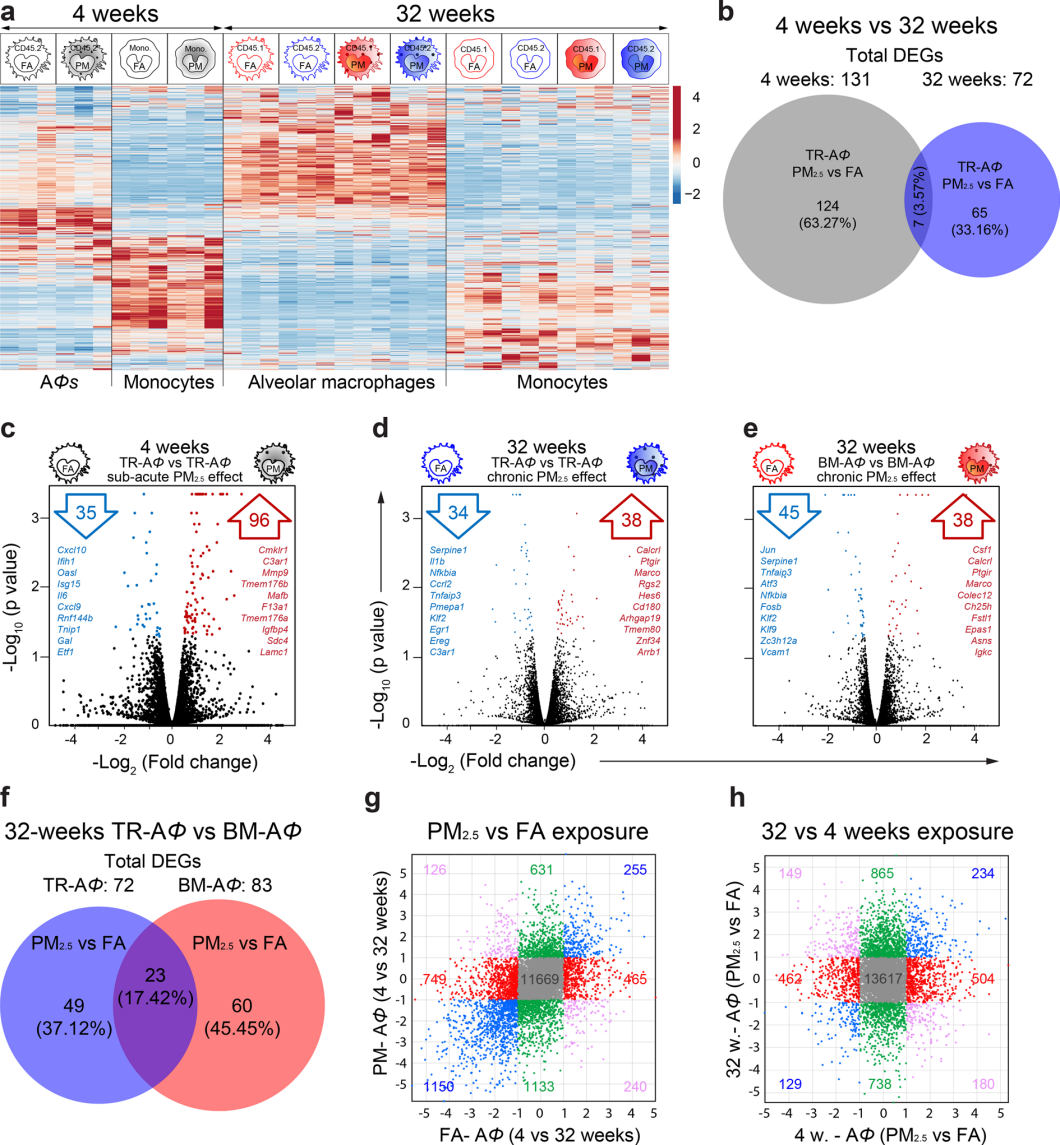
Transcriptomic landscape of A*Φ* with PM_2.5_ exposure. (**a**) Heat map of all significantly differentially expressed genes in A*Φ* and lung monocytes, of CD45.2 and CD45.1 origin from 4- and 32-weeks of FA and PM_2.5_ exposed mice. (**b**) Quantitative Venn diagram showing unique and common DEGs at 4- and 32-weeks of exposure. (**c–e**) Volcano plots of different pairwise comparisons showing, up- (red) and downregulated (blue) genes. In each volcano plot, top listed genes are ten Hallmark genes and/or immunological signature genes. (**f**) Quantitative Venn diagram showing unique and common DEGs in TR-A*Φ* and BM-A*Φ* at 32-weeks exposure. (**g, h**) Common and unique significant DEGs in four-way plots showing the effect of (**d**) PM_2.5_ vs FA exposure, and (**e**) exposure time (4- and 32-weeks). Fold change (Log_2_) values are plotted for respective comparisons mentions on the x- and y-axes. Data were generated using three mice from each group.

Lung A*Φ* identified as CD64^+^CD11c^hi^CD11b^lo^ and monocytes identified as Ly6G^–^MHC II^–^CD64^lo^CD11b^hi^, (of both the CD45.2^+^ and CD45.1^+^) were isolated using fluorescence-activated cell sorting (FACS) from lungs of 4- and 32-weeks exposed mice and three mice from each group (FA and PM_2.5_ groups of both the 4- and 32-weeks exposure) were randomly selected for RNA-sequencing. We sequenced A*Φ* and monocytes of both CD45.1 and CD45.2 origin at 32-weeks while only CD45.2 origin cells could be sequenced at 4-weeks as sorted BM-A*Φ* numbers at 4-weeks did not yield enough RNA quantity. Monocytes and A*Φ* samples clustered separately in the principal component analysis (PCA plot) with a variance of 43.8% in PC1 and 4- and 32-weeks samples showing less variance, but separated in PC2 (Supplementary Fig. 6a). Differentially expressed significant genes (DEGs) from lung A*Φ* and monocytes at 4-weeks (only CD45.2 origin) and 32-weeks (both CD45.1 and CD45.2 origin), were compared. This analysis revealed unique and widespread differences in the expression profile of genes at these time points (4- and 32-weeks) with a distinctive expression profile in the A*Φ* and monocyte populations (Fig. 4a).

#### Differential impact of PM_2.5_ in TR-A*Φ*

In response to PM_2.5_ at 4-weeks, TR-A*Φ* displayed a unique transcriptomic signature (131 genes), sharing only seven DEGs at 32-weeks exposure (Fig. 4b–d, and Supplementary Fig. 6e, f). Genes that are known to play a crucial role in maintaining lung immune defense and homeostasis *viz*. macrophage scavenger receptor genes (CD163, Marco), matrix metalloproteinase (Mmp9), Sftpc (surfactant associated protein C), chemokines (Cxcl13, CCL9), complement genes (C1qb, C1qc, Cfh, C3ar1) and heat shock protein family genes (Hsp2, Hsph1, Hspa1a, etc.) were found to be upregulated, while chemokines (CXCL9/10) and several proteins involved in anti-viral immunity (Oasl, Ifih1, Isg15, etc.) were found to be downregulated. The associated significant GO terms were related to immune system processes, defense response, and inflammatory response (Fig. 4c and Supplementary Table 2).

PM_2.5_ exposure induced distinct transcriptomic responses in TR-A*Φ* and BM-A*Φ* at 32-weeks and 49 and 60 unique DEGs in TR-A*Φ* and BM-A*Φ* respectively were identified (Fig. 4d–f and Supplementary Table 2). TR-A*Φ* shared 23 DEGs with BM-A*Φ* at 32-weeks of PM_2.5_ exposure (Fig. 4f) and among these, Fstl1, CXCR1, and Marco were found to be upregulated in both the A*Φ*, suggesting a possible commonality of homeostatic response. Fstl1 for instance, promotes airway remodeling in airway inflammatory diseases^12^, while CXCR1 is known to bind CXCL6 in addition to CXCL8, with the CXCL8-CXCR1 axis activates multiple signaling pathways controlling proliferation and differentiation of cells^13,14^. Indeed the enriched GO terms associated with DEGs in TR-A*Φ* at 32-weeks comprised of the inflammatory response, myeloid leukocyte migration, and regulation of cell proliferation (Fig. 4d–f and Supplementary Table 2). (The overall effect of PM_2.5_ exposure on TR-A*Φ* in 4 weeks and 32 weeks exposure are summarized in Supplementary Fig. 8).

#### Differential impact of PM_2.5_ and aging in A*Φ*

To understand aging-related versus PM_2.5_ effects, we initially compared the transcriptomic profile of TR-A*Φ* derived from FA exposed mice at 4- and 32-weeks. We found 750 unique DEGs, that were most likely age-related changes. (Supplementary Fig. 6b). These results suggested an upregulation in the processes associated with cellular metabolism and downregulation of the immune system and immune defense process (Supplementary Table 2). Comparison of the transcriptomic profile of TR-A*Φ* derived from PM_2.5_ exposed animals at 4- and 32-weeks identified 976 DEGs which represent PM_2.5_ effect superimposed on aging (Supplementary Fig. 6c). Moreover, when comparing the FA-FA and PM_2.5_-PM_2.5_ groups, 429 DEGs were common in 32-weeks and 4-weeks comparison (Supplementary Fig. 6d). Therefore, to identify the DEGs that were exclusive and/or common to PM_2.5_ and aging we did four-way correlation analysis. First, we compared the A*Φ* transcriptome at 4- and 32-weeks in FA with 4- and 32-weeks PM_2.5_ exposure (Fig. 4g). We found 1,214 DEGs (749 + 465) that were regulated only in FA, but showing no change in PM_2.5_, suggesting possible aging-related regulated genes; 255 genes were found to be upregulated with both the PM_2.5_ and FA. Comparing 32-weeks (FA vs PM_2.5_) with 4-weeks (FA vs PM_2.5_) exposure identified 865 genes upregulated only in 32 weeks PM_2.5_ exposure, 504 genes upregulated only in 4-weeks exposure and 234 genes that were upregulated at both the time points (Fig. 4h). Overall this analysis helped to identify PM_2.5_ specific and aging-associated genes in A*Φ*.

### A mixed inflammatory response by A*Φ* in response to chronic PM_2.5_ exposure

**Figure 5:**
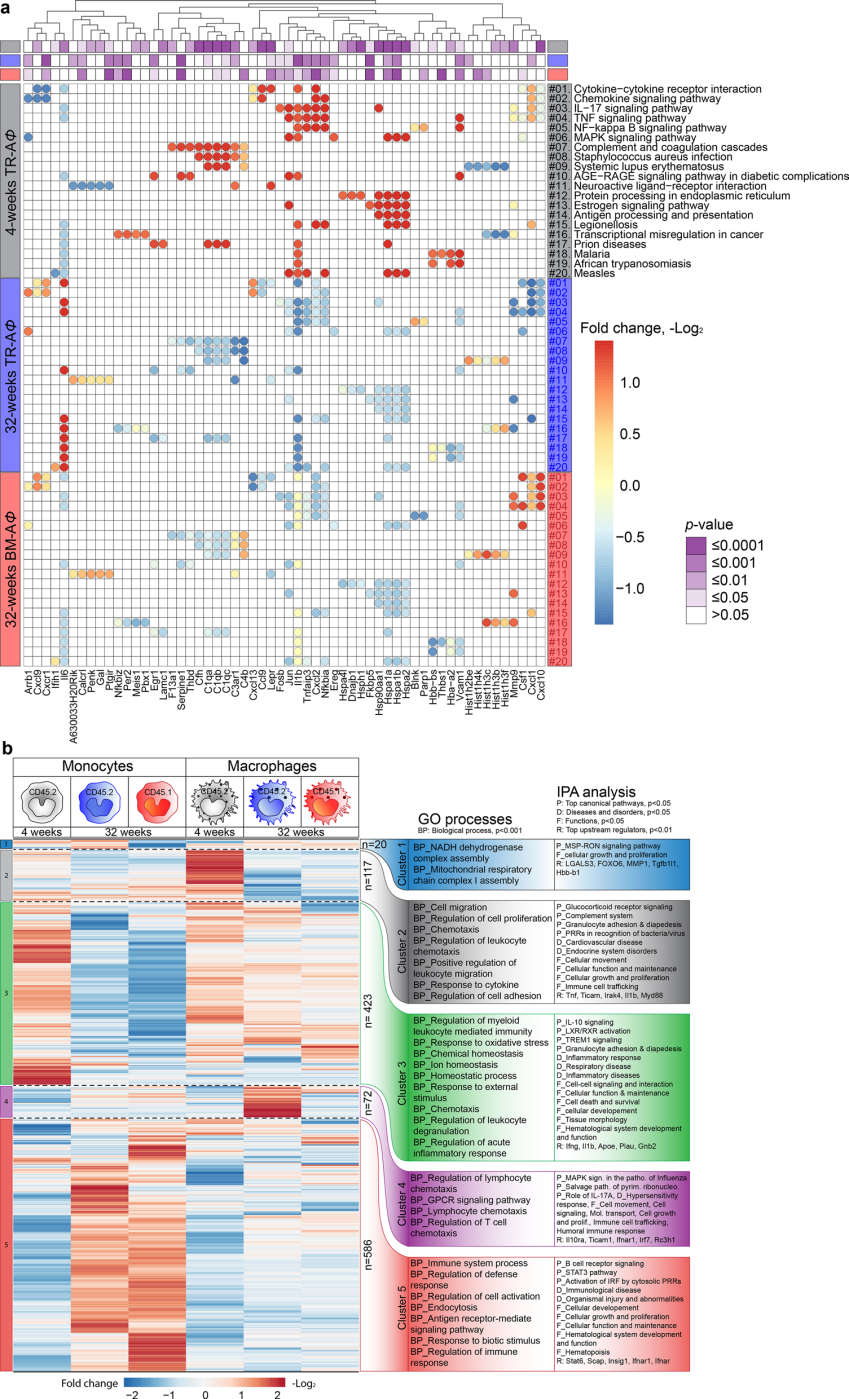
Gene ontology processes and pathways associated with differentially expressed genes. (**a**) Heatmap of enriched pathways and associated genes at 4- and 32-weeks of PM_2.5_ exposure. (**b**) Heatmap (Log_2_ fold change, K-means clustering) of common DEGs in monocytes and A*Φ* from 4- and 32-weeks PM_2.5_ exposed mice. GO biological process (*p* < 0.001) and IPA analysis results corresponding to each cluster genes are listed on the right of the heatmap, (P: Top canonical pathways, D: diseases and disorders, F: top biological, cellular functions, R: top upstream regulators). Data were generated using three mice from each group. (see also Supplementary Fig. 7).

Gene ontology (GO) biological processes associated with DEGs at 4- and 32-weeks in TR-A*Φ* were identified using TopGO analysis; GO term redundancy was removed using REVIGO^15^. (Supplementary Fig. 7a). At 4-weeks, identified GO terms were related to stress response, cytokine response, and immune system processes, while at 32-weeks the associated GO processes corresponded with tissue remodeling, cell signaling, system development, oxidative stress, macrophage differentiation (Supplementary Fig. 7a). Pathway analysis revealed differentially regulated pathways at 4- and 32-weeks (Fig. 5a). Cellular pathways that are most likely involved in immediate defense such as complement and coagulation cascade, NF-kappa B signaling pathway, *Staphylococcus aureus* infection, antigen processing, and presentation were specifically enriched at 4-weeks. At 32-weeks the genes involved in NF-kappa B signaling pathway were found to be downregulated. Cytokine-cytokine receptor interaction, chemokine signaling pathway, IL-17 signaling pathways were enriched at both the 4- and 32-weeks TR-A*Φ*, but with unique genes at each time point, indicating a potentially unique temporal regulation of inflammation and resolution in response to PM_2.5_ (Fig. 5a).

In order to determine pathways distinctively regulated by PM_2.5_ in lung monocytes and macrophages, regardless of their origin (CD45.1 or CD45.2), we compared, DEGs (1,218 DEGs) that were common among CD45.1 and CD45.2 monocyte and macrophage populations at 4 to 32-weeks. K-means clustering yielded 5-clusters, with functionally different DEGs (Fig. 5b). GO terms were assigned to each cluster using GO enrichment analysis^16–18^ and the networks, functional analyses, pathways, etc. were generated through the use of IPA (QIAGEN Inc., https://www.qiagenbioinformatics.com/products/ingenuity-pathway-analysis) (Fig. 5b)^19^. Cluster 1 was the smallest cluster comprised of only 20 genes and enriched GO processes were associated with mitochondrial function. Cluster 2 (117 genes) DEGs, upregulated in 4 weeks in A*Φ*, were associated with cell migration, proliferation, and chemotaxis process with IPA analysis suggesting pathways involved in signaling, complement system, granulocyte adhesion & diapedesis and pattern recognition receptors (PRRs); enriched functional components were cellular movement, cellular function and maintenance, growth and proliferation and immune cell trafficking. Cluster 3 (423 genes) DEGs that are relatively upregulated at 4-weeks exposure (CD45.2 monocytes and A*Φ*) were associated with the homeostatic process, response to oxidative stress and regulation of acute inflammatory response, and pathways and included IL-10 signaling and activation of liver X receptors and retinoid X receptors (important regulators of macrophages and key players in inflammatory and metabolic disorders)^20^ and TREM1 signaling. TREM1 (triggering receptor expressed on myeloid cells-1) expressed on most of the innate immune cells, known to amplify inflammatory response upon activation^21,22^. Cluster 4 (72 genes) DEGs were upregulated only in TR-A*Φ* at 32-weeks and associated GO processes were predominantly lymphocyte chemotaxis, with corresponding pathways (MAPK signaling, salvage pathways of pyrimidine ribonucleotides and role of IL-17, molecular transport, cellular growth, and proliferation, immune cell trafficking and humoral immune response) indicating a distinct pro-inflammatory shift in response to PM_2.5_ in TR-A*Φ* at 32 weeks. Cluster 5 (586 genes, the largest cluster), DEGs were uniquely upregulated in monocytes (CD45.2 and CD45.1) at 32-weeks (when compared to macrophages). Corresponding GO terms were related to immune system processes, defense response, etc. indicating a delayed pro-inflammatory shift of infiltrating bone marrow-derived monocytes and also indicating a reorganization in the inflammatory profile of TR-A*Φ* in the lung (Fig. 5b).

## Discussion

**Figure 6:**
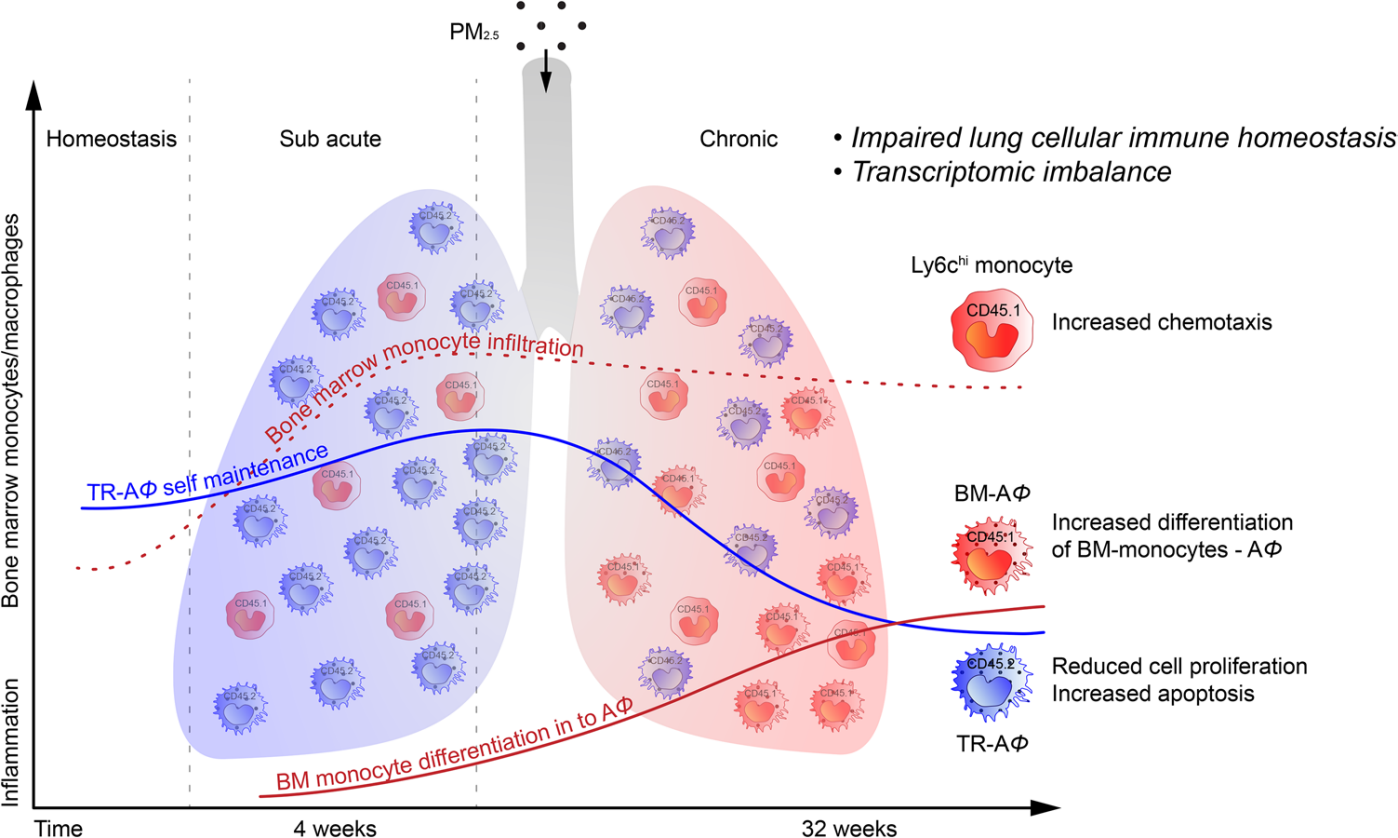
Schematic illustration of key changes in lung inflammation in acute and chronic PM_2.5_ exposure. The figure summarizes the key finding of the PM_2.5_ exposure for 4-weeks showing the increased infiltration of Ly6c^hi^ monocytes and at 32-weeks showing impaired lung cellular homeostasis.

Our study demonstrates several important new findings on how the innate immune response in the lungs is altered by chronic real-world exposure to PM_2.5_. We employed a unique lung shielded chimeric mice model to identify the differential contribution of hematopoietic cells migrating to the lung, in response to the temporal scale of PM_2.5_ exposure. Our findings suggest the following (Fig. 6): 1. Hematopoietic cells are seen infiltrating the lung by 4-weeks and continuing until 32-weeks in response to PM_2.5_ exposure. 2. Donor BM (CD45.1) derived cells contribute to the overall A*Φ* pool, but only with chronic exposure (32-weeks), coinciding with an imbalance in self-renewal capacity and enhanced apoptosis of TR-A*Φ*. 3. The incorporation of BM-*Φ* to the overall A*Φ* pool is associated with an inflammatory shift in the protein-coding transcripts 4. Chronic PM_2.5_ exposure resulted in a reduction in alveolar surface area/gas exchange.

Bone marrow chimeras and monocyte reporter mice have been fundamental to our understanding of the role of monocytes as they differentiate into immature macrophages and ultimately into mature alveolar macrophages^4^. A large part of our understanding of the mechanisms of acute and chronic inflammatory lung disease however have been derived from short term experimental models, that rely on acute high-dose exogenous exposures (ozone, LPS, or bleomycin). There have only been a limited number of studies that have evaluated the contribution of the bone marrow-derived cells to lung inflammation in response to air pollution, with these studies relying exclusively on intratracheal exposures and/or short durations of exposure^23–25^. It is fair to conclude that the true impact of a pervasive omniscient stimulus (like air pollution) cannot be adequately simulated or inferred in these models. There is a need to better understand the biology of the A*Φ* population in the lung, a unique finite subset of cells developing from the yolk sac that self-renew and do not depend on adult monocytes for maintenance^3,6,26^. The response of this population to a chronic ongoing inflammatory insult has not been studied, and in particular if peripheral monocytes play a role in mitigating loss-of-resident macrophage subsets following injury has yet to be satisfactorily addressed^27^.

In our study, PM_2.5_ exposure for 4-weeks resulted in the recruitment of bone marrow-derived Ly6c^hi^ monocytes. Both monocyte chemoattractant subfamily members, CCL7 (monocyte-chemotactic protein 3/MCP3) and CCL2 (MCP1) were found to be upregulated in A*Φ* at 4-weeks in response to PM_2.5_^28–30^. This data suggest a possible egress of inflammatory monocytes from BM with short term PM_2.5_ exposure in response to these chemokines.

While bone marrow-derived monocytes have been shown to differentiate into A*Φ* phenotype in vitro, it is only recently, that they have been shown to play a role in differentiating to TR-A*Φ* in the lung following bleomycin-induced injury, facilitating sustained lung inflammation and fibrosis^4^. In contrast to the role of low-grade proliferation in the maintenance of A*Φ* levels, adult monocytes contribute to the A*Φ* population during inflammation over longer time frames of exposure especially given ongoing apoptosis of TR-A*Φ* in the lung. At 4-weeks in response to PM_2.5_, A*Φ*s were primarily of tissue-resident origin. Siglec F expression on i*Φ* was increased with PM_2.5_ at 4-weeks, suggesting maturation of this population towards A*Φ*. In contrast, we observed apoptosis and impaired self-renewal in TR-A*Φ* with PM_2.5_ exposure at 32-weeks, which may have facilitated the influx of bone marrow-derived monocytes that then differentiate into macrophages. Expression of Siglec F, the characteristic marker for A*Φ,* on these BM-A*Φ* was indeed comparable to TR-A*Φ* in 32 weeks PM_2.5_ exposed mice.

The incorporation of BM-*Φ* to the overall A*Φ* pool was associated with an inflammatory reorganization in the protein-coding transcripts in both TR-A*Φ* and BM-A*Φ*. Multiple scavenger receptors were differentially upregulated in response to chronic PM_2.5_ exposure at 32 weeks. CD163 is a macrophage-specific scavenger receptor and its upregulation in A*Φ* indicates an attempt at phagocytosis of ongoing inhaled particles^31^. CD200, an inhibitory receptor that plays a critical role in maintaining lung immune homeostasis was downregulated at 4-weeks but upregulated at 32-weeks, indicating a balance between shifting priorities of responding to an inflammatory external trigger acutely, and switching to a homeostatic defense mode chronically^32^.

Chronic PM_2.5_ exposure induced marked inflammatory polarization and reduction in alveolar surface area/gas exchange. Thirty-two weeks of PM_2.5_ exposure resulted in a decrease in L_M_, a surrogate for gas exchange surface of the lung alveoli consistent with epidemiological reports of showing an association between chronic PM_2.5_ exposure, reduced lung function parameters, (COPD) and lung fibrosis, etc. in humans^33–37^. Although lung fibrosis with PM_2.5_ has been recently reported by other groups, this has been with intranasal dosing or with agents such as bleomycin^38,39^. In our study the upregulation of TGF-β along with CXCL13 in TR-A*Φ* with 32-weeks of PM_2.5_ exposure provides a possible mechanistic pathway of increased fibrosis. CXCL13, a chemokine that controls the trafficking of B cells has been recently demonstrated to be directly linked to pulmonary fibrosis^40,41^ was also upregulated in PM_2.5_ group at 4-weeks, and further increased by two-fold in TR-A*Φ* at 32-weeks.

Using a four-way analysis of the transcriptomic data (Fig. 4g, h) we identified unique gene sets specific to PM_2.5_ exposure versus that of age-related changes. Immune senescence in the elderly has been well defined as ‘inflammaging’, which refers to elevated levels of tissue and circulating pro-inflammatory cytokines in the absence of an immunological threat^42^. Moreover, it has been reported that aging a state of irreversible inhibition of cell proliferation also changes the cellular secretory profile^43^. Indeed we found differential expression of pro and anti-inflammatory genes as well as expression of cytokines and chemokines at both 4- and 32-weeks of PM_2.5_ exposure.

In conclusion, our findings suggest a time-dependent PM_2.5_ entrainment of a BM-derived monocytes infiltration that together with enhanced apoptosis of TR-A*Φ* and a reorganization in profile of inflammatory genes may contribute to altered pulmonary immune homeostasis and perpetuation of chronic inflammation. Our study to the best of our knowledge is the first comprehensive chronic exposure study to demonstrate the recruitment of BM-A*Φ* and increased apoptosis in TR-A*Φ* in the lung in response to chronic exposure to real-world PM_2.5_. There are limitations to our study including lack of measures of lung function and other immune cell types in the lungs such as alveolar type II, lymphocytes, DCs, etc. Our findings have implications for human health given known observations in prospective observational cohort studies^44,45^ that suggest an important role for air pollution exposure in impaired lung function.

## Methods

### Mice

C57BL6/J (CD45.2, stock no: 000664) and B6.SJL-Ptprc^a^ Pepc^b^/BoyJ (CD45.1, stock no: 002014) 6–8 weeks old male mice were procured from *The Jackson Laboratory.* All animal experiments were done according to the guidelines and were approved (Protocol number 2016-0319) by the Institutional Animal Care and Use Committee at Case Western Reserve University, Cleveland.

### Generating lung shielded chimeric mice

To distinguish the tissue-resident alveolar macrophages (TR-A*Φ*s) and bone marrow-derived alveolar macrophages (BM-A*Φ*s) in the lungs, lung shielded chimeric mice were generated as described by elsewhere^4^. In brief, 7–8 weeks old, males, C57BL/6J recipient mice (now onwards CD45.2) were lethally irradiated (single dose of 1,000 cGy, γ-radiation) using^137^Cs radioactive source. Mouse chest was shielded with a lead block to protect the TR-A*Φ*s from radiation (Supplementary Fig. 1a), 6 h. post-irradiation, “busulfan” (30 mg/kg) was administered via intraperitoneal route, followed by bone-marrow transplantation (24 h. after irradiation) from age and sex-matched B6.SJL-Ptprc^a^ Pepc^b^/BoyJ donor mice (now onwards CD45.1). Mice were housed and maintained in SPF conditions at Case Western Reserve University. Eight weeks after bone marrow transplantation 50–100 µl of blood was collected from mice and examined for the monocyte chimerism in peripheral blood (proportions of CD45.1 and CD45.2 cells). Chimeras having > 97% circulating monocytes of donor origin (CD45.1) were used for experiments (Supplementary Fig. 1b). The naïve shielded chimeras have > 99% of lung TR-A*Φ* of CD45.2 origin while > 97% peripheral blood monocytes of CD45.1 origin, therefore, discriminate BM-A*Φ* from TM-A*Φ*.

### PM_2.5_ exposure model

To mimic real-world chronic particulate matter air-pollution exposure on lung inflammation, we placed mice in chambers connected to the Versatile Aerosol Concentrator and Enrichment System (VACES) available in our lab. This system allows us to assess the in vivo effects to “real world” particles, at relevant concentrations, circumventing limitations inherent to intra-nasal and intra-tracheal exposure^7,8,46^. Our Versatile Aerosol Exposure Concentrator System (VACES) in downtown Cleveland, provides 8–10 × concentration over ambient exposure concentrations and is identical to daily PM_2.5_ concentrations in Beijing or New Delhi (typically between 70 and 100 µg/m^3^). Mice were placed in VACES chambers to expose to concentrated PM_2.5_ or filtered air (FA) for control, for 6–8 h/day, 5 days/weeks for 4–32 weeks. Post-exposure mice were euthanized by lethal sedation with isoflurane inhalation and bronchoalveolar lavage fluid (BALF) and blood were collected. Mouse whole-body perfusion was done with ice-cold PBS through the left ventricle, and lungs, spleen, and bone marrow were collected for further analysis.

The weekly average ambient PM_2.5_ mean concentration for 4- and 32-weeks exposures were 10.79 ± 1.4 and 90.09 ± 24.18 µg/m^3^ respectively. The total dose of PM_2.5_ inhaled during the 4- and 32-weeks exposures corresponded to 15.66 ± 1.63 and 138.89 ± 11.79 μg respectively, assuming a ventilation rate of 105 breaths/min. and 0.2 cc/breath in mice.

### Flow cytometry

The macrophages, monocytes, and neutrophils in the lungs and monocyte in the blood, spleen, and bone marrow were analyzed by flow cytometry using cell-specific surface markers and specific gating strategies for each tissue.

#### Lung

lung tissue was enzymatically digested/dissociated into single-cell suspension using mouse “Lung Dissociation Kit” and “gentleMACS Octo Dissociator with Heaters” both from Miltenyi Biotech Inc. as per the manufacturer’s instructions. In brief—lungs were washed twice with cold PBS, cut into small pieces, transferred to MACS ‘C Tube’ containing the enzyme mix. The tube then loaded on the gentleMACS instrument and run the lung-specific program. The dissociated cell suspension was passed through a 70-µm cell strainer and washed three times with cold PBS containing 1% FBS and 0.2% BSA.

Cells were washed three timed with protein-free PBS and stained with viability dye (Live/DEAD fix blue, Thermo Fisher Scientific) in the dark, cells were washed again and incubated with FcBlock (anti-mouse CD16/CD32) in FACS buffer, followed by staining with fluorochrome-conjugated antibodies for Lung Panel (antibodies, clones, fluorochromes, and manufacturers were described in Supplementary Table 1). Fluorescence minus one controls were used to set the gates to select the positive population. Lung A*Φ*s were identified as CD64^+^CD11c^high^CD11b^lo^ (or by CD11c^high^Siglec-F^high^) and i*Φ*s as CD64^+^CD11c^low^CD11b^hi^ (Supplementary Fig. 4)^2^.

#### Spleen

Spleens were placed in 70-µm cell strainer and gently rubbed used syringe plunger head to get the single-cell suspension, incubated with RBC Lysis buffer (Biolegend Cat: 420301) and washed three times with PBS containing 1% FBS and 0.2% BSA. Cells were counted and 1–2 million cells were used for flow cytometry staining using the specific panel for spleen (Supplementary Table 1 and Supplementary Fig. 4).

#### Blood and bone marrow

Blood was collected from inferior vena cava or directly from heart, into EDTA-coated tubes, plasma was collected (2000* g* 15 min, 4 °C), stored at -80 °C, and RBC was lysed using RBC lysis buffer (Biolegend Cat: 420,301). For bone marrow, femurs and tibia were flushed and marrow was resuspended in 3–5 ml cold RPMI containing 5% FBS, and RBC lysis was performed. After the lysis, cells were washed, counted, and used for flow cytometry. The same set of antibodies were used for blood and bone marrow (Supplementary Table 1 and Supplementary Fig. 4).

Blood, bone marrow, and spleen cells were acquired on the BD LSR II flow cytometer (antibody panels are described in Supplementary Table 1 and Supplementary Fig. 4). Lung cell sorting was performed on FACSAria II SORP (BD), using an 80-µm nozzle. A minimum of 50–80 thousand live cells (A*Φ* and monocytes of CD45.1^+^ and CD45.2^+^) were isolated and used for RNA-seq analysis. Flow cytometry data were analyzed using FlowJo software v10 (BD).

### BrdU labeling of A*Φ* in vivo

BrdU was administered intratracheally using endotracheal intubation kit from Kent Scientific as per the manufacturers’ instructions. In brief, mice were anesthetized with isoflurane inhalation and monitor for stable breathing during anesthesia. BrdU (1.63 micromoles of BrdU/mouse in 50 µl PBS/day for 3 days) was administered directly in the lungs using 22 g cannula through the trachea. After the successful delivery of BrdU, mice were monitored until recovery and return to normal housing. Mice were euthanized 24 h after the last BrdU delivery and lungs and other tissues were collected for analysis.

### Histological staining and lung morphometry

After euthanasia blood was collected and lungs were infused through the trachea with 4% paraformaldehyde at 25 cm H_2_O pressure for 10 min and tied off and placed inflated in formalin for 2 days. The heart was excised and lung volumes (V_L_) were measured by water displacement. For histology, the left lung was dehydrated in ethanol and embedded in the O.C.T. compound and cut into 5-*μ*m sections. Lung sequential sections were stained with hematoxylin and eosin (H&E) for morphometry analysis, Masson’s trichrome staining for fibrosis, F4/80 for macrophages and with cleaved caspase 3 for apoptosis.

The primary lung function i.e. the gas exchange is critically dependent on the availability of the surface area of the interface establishing contact between the air in the alveoli and the blood in the alveolar capillaries. This can be quantified using physiological equations for predicting oxygen uptake^9^.

The lung sections were acquired at 4 × using the Keyence microscope, multiple photographs were taken (with a 10% overlap) to cover the whole lung section. These images were later stitched together (using Keyence microscope software) to reconstruct a single image of the entire section. An additional set of twenty randomly chosen areas from each lung section was photographed with the 20 × lens. Both of these image sets (4 × reconstructed single image and 20 random images of 20 ×) were used for lung morphometric quantitation using STEPanizer v1.0 as described^10,11,47^. In brief, parenchymal fraction (F_P_) and parenchymal volume (V_P_ = F_P_ × V_L_) were measured using 10 × images. The lung volumes (V_L_) used for calculating parenchymal volumes (V_P_) were measured in the fixed lungs by water displacement. Parenchymal tissue fraction (F_PT_), the alveolar fraction (F_ALV_), L_M_, and alveolar surface area (S_A_) were measured using 20 × images with short sampling grid line segments (of length d). The mean linear intercept of parenchymal airspaces (L_M_) was calculated as d Pair/P intercepts, and S_A_ was then equal to 4 Vp/L_M_. These L_M_ and alveolar surface area calculations are based on robust theory and proper stereological measurements.

### RNA-sequencing and bioinformatics analysis

Lung A*Φ* and monocytes population (of CD45.1 and CD45.2 origin) were isolated using fluorescence-activated cell sorting (BD FACSAria II SORP instrument) from FA and PM_2.5_ exposed mice at 4- and 32- weeks’ time points. Cells were collected in RPMI 1,640 (containing 20% FBS, 3 mM EDTA), centrifuged and RNA was extracted using RNeasy Plus Micro Kit (QIAGEN cat: 74034) and eluted in 15 µl of deionized water for subsequent transcriptomic analysis. For each condition and time point, three biological replicates were used. Libraries for RNA-seq were prepared using the SMARTER kit from Takara and RNA sequencing was carried out at DNA sequencing core, University of Michigan Medical School, Michigan, with the paired-end, 50 million reads.

Sequencing reads generated from the Illumina platform were assessed for quality and trimmed for adapter sequences using TrimGalore! v0.4.2 (Babraham Bioinformatics), a wrapper script for FastQC and cutadapt. Reads that passed quality control were then aligned to the mouse reference genome (mm10) using the STAR aligner v2.5.1^48^. The alignment for the sequences was guided using the GENCODE annotation for mm10. The aligned reads were analyzed for differential expression using Cufflinks v2.2.1^49^, an RNASeq analysis package that reports the fragments per kilobase of exon per million fragments mapped (FPKM) for each gene. Differential analysis report was generated using the cuffdiff command performed in a pairwise manner for each group. Differential genes were identified using a significance cutoff of q-value < 0.05 (Benjamini Hochberg FDR corrected for multiple testing correction). The genes were then subjected to gene set enrichment analysis (GenePattern, Broad Institute) to determine any relevant processes that may be differentially over-represented for the conditions tested.

Quantitative Venn diagrams were created using a web application named ‘BioVenn’ available at https://biovenn.nl/^50^ Hallmark gene set^51^ and Immunologic signature genes^52^ were identified using Molecular Signatures Database v7.0 (MSigDB) accessible at https://software.broadinstitute.org/gsea/msigdb/index.jsp, Gene Set Enrichment Analysis (GSEA)^51,53,54^.

### Pathway analysis and Go analysis

The Data (significantly impacted pathways, biological processes, molecular interactions, miRNAs, SNPs, etc.) were analyzed using Advaita Bio’s iPathwayGuide (https://www.advaitabio.com/ipathwayguide). This software analysis tool implements the ‘Impact Analysis’ approach that takes into consideration the direction and type of all signals on a pathway, the position, role and type of every gene, etc., as described in^55–58^. The data obtained were corrected for multiple comparisons using the Bonferroni test and a significance cutoff of *p *value < 0.05 was considered as statistically significant.

### Statistical analysis

Statistical details of each experiment including the descriptions of samples (mice) and statistical test are mentioned in each figure legend. The number of mice used is shown in the figures. GraphPad Prism 8.3 software (GraphPad Software) was used to analyze and plot the data. Data were shown as the mean ± SEM. Differences were analyzed using two-tailed Student’s t-tests and one-way ANOVA with Bonferroni’s posthoc test for multiple comparisons and were considered significant when the *p *value ≤ 0.05 and exact *p* values are mentioned in figures for respective comparisons. Statistical analysis for RNA-Seq described above in “RNA-sequencing and bioinformatics analysis”.

## Study design

To effectively distinguish the lung tissue-resident alveolar macrophage (TR-A*Φ*), from bone-marrow-derived macrophages (BM-A*Φ*), lung shielded chimeric mice were generated (in which the lung resident cells were CD45.2 origin and circulating peripheral cells were of CD45.1 origin). Mice were placed in air pollution exposure chambers to study the impact of acute and chronic life-long PM_2.5_ exposure on lung inflammation. Mice were exposed to 4–32 weeks to filtered air (FA) or concentrated ambient PM_2.5_ air (PM_2.5_) and lung A*Φ* and monocytes of CD45.1 and CD45.2 origin were quantified and sorted using flow cytometry (FC) and used for RNA-seq analysis. Monocytes in the peripheral blood, spleen, and bone marrow were also quantified using FC. Bioinformatic analysis of the gene expression profile of lung macrophages and monocytes were carried out to get the insight of the transcriptomic map of lung macrophage in acute and chronic PM_2.5_ exposure.

## Supplementary information

Supplementary table 1.

Supplementary figures.

Supplementary table 2.

## Data Availability

The datasets generated during the current study are submitted in the GEO, the accession number is GSE143787 and can be assessed at https://www.ncbi.nlm.nih.gov/geo/query/acc.cgi?acc=GSE143787 using reviewers’ token “qdkxgwqgprwvtun” (without quotes).
