# Supplementary material for: Differential contribution of bone marrow-derived infiltrating monocytes and resident macrophages to persistent lung inflammation in chronic air pollution exposure
Source: Sci Rep. 2020 Sep 1;10:14348. doi: 10.1038/s41598-020-71144-1 (PMC7462977; doi:10.1038/s41598-020-71144-1)
Supplement: Supplementary file 2 — Supplementary figures. [file 41598_2020_71144_MOESM2_ESM.docx]

# Differential contribution of bone marrow-derived infiltrating monocytes and resident macrophages to persistent lung inflammation in chronic air pollution exposure

Roopesh Singh Gangwar, Ph.D.,^1^ Vinesh Vinayachandran, Ph.D.,^1^ Palanivel Rengasamy, Ph.D.,^1^ Ricky Chan, Ph.D.,^2^ Bongsoo Park, Ph.D.,^3^ Rachel Diamond-Zaluski, B.Sc.,^1^ Elaine Ann Cara, M.Sc.,^1^ Anthony Cha, B.Sc.^1^ Lopa Das, Ph.D.,^1^ Courteney Asase, M.A.,^1^ Andrei Maiseyeu, Ph.D.,^1^ Jeffrey Deiuliis, Ph.D.,^1^ Jixin Zhong, Ph.D.,^1^ Wayne Mitzner, Ph.D.,^4^ Shyam Biswal, Ph.D.,^3^ and Sanjay Rajagopalan, M.D.,^1*^

^1^Case Cardiovascular Research Institute, Case Western Reserve University, Cleveland, OH 44106 USA

^2^Department of Environmental Health and Engineering, Johns Hopkins University School of Public Health, Baltimore, MD 21205

^3^Case Western Reserve University, Cleveland, OH 44106 USA

**^*^Corresponding author**

Sanjay Rajagopalan, MD, FACC, FAHA

Chief, Division of Cardiovascular Medicine, University Hospitals

Harrington Heart and Vascular Institute (HHVI),

Director, Case Cardiovascular Research Institute

Herman K. Hellerstein MD, Professor of Cardiovascular Research

Professor, Department of Internal Medicine and Radiology

Case Western Reserve University

11100 Euclid Ave, Cleveland, OH 44106; Mailstop: 5038

Phone: 216-844-5125 | Fax: 216-844-8318

e-mail: [sxr647@case.edu](mailto:sxr647@case.edu),

# Supplementary Figures


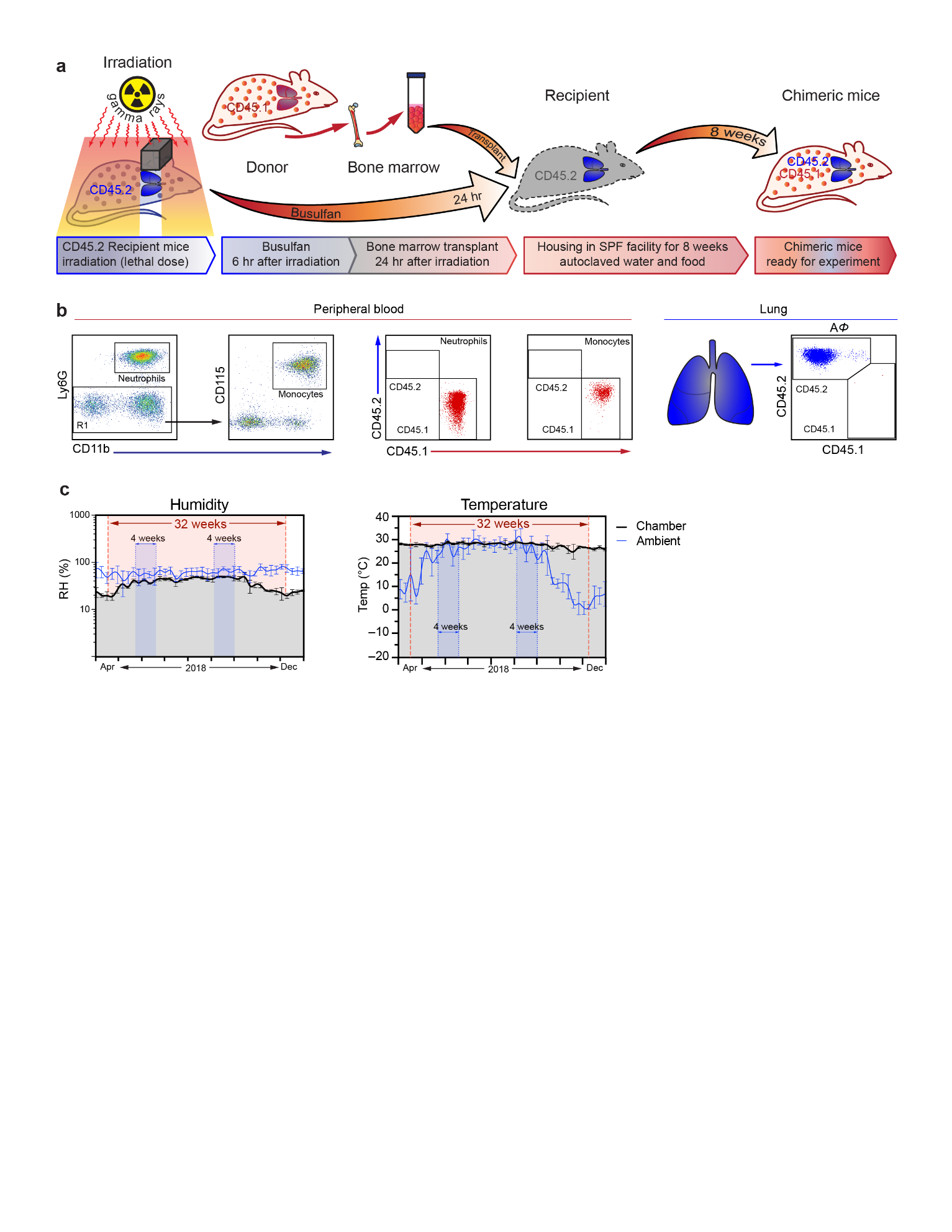


## Supplementary Figure 1. Development of lung shielded chimeric mice model.

**a.** Schematic representation of generation of lung shielded chimeric mice. **b.** Representative flow cytometry plots from a naïve chimeric mouse, showing peripheral blood neutrophils and monocytes of CD45.1 origin and lung A*Φ* are of CD45.2 origin. **c.** Humidity and temperature data from ambient and VACES chambers during the 4 and 32-weeks of exposure time.


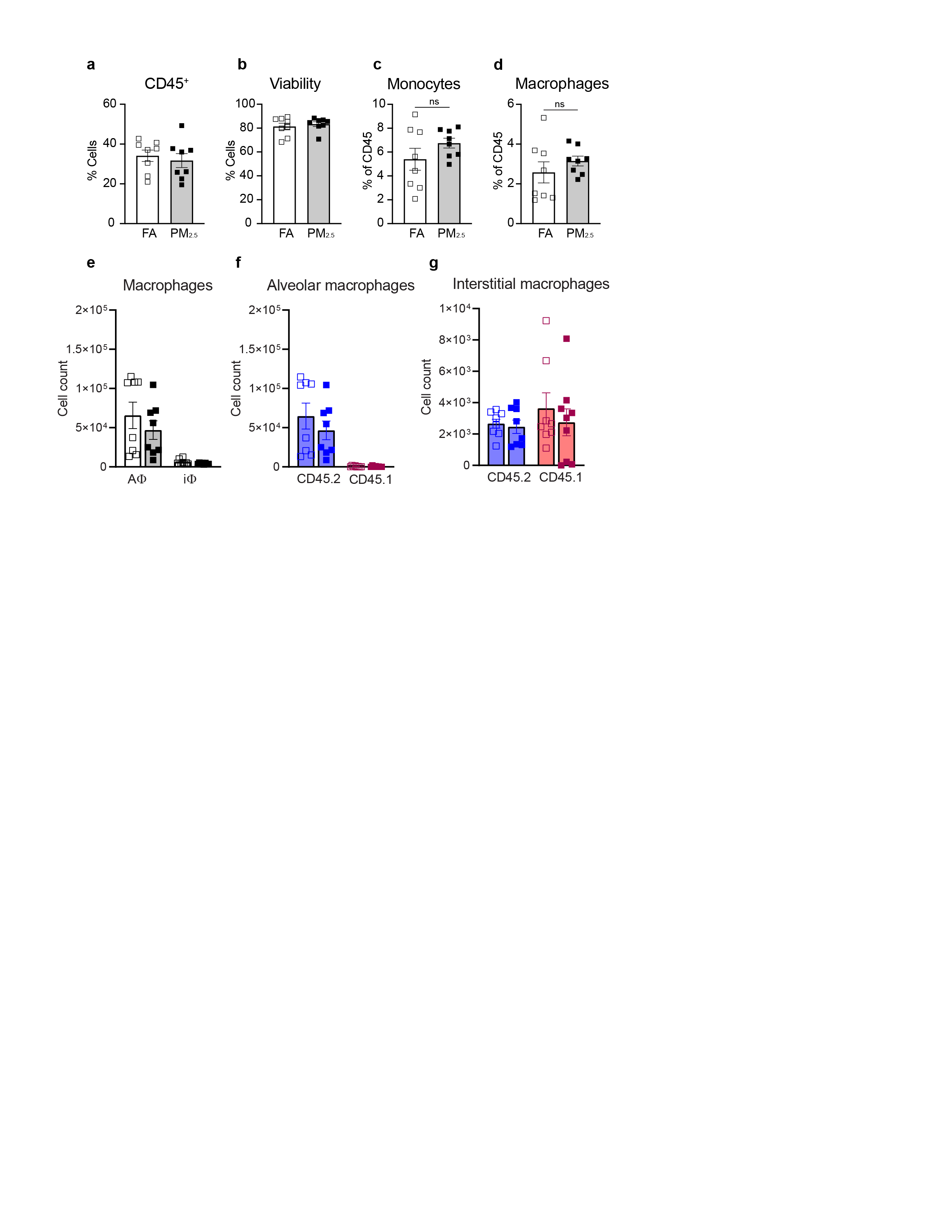


## Supplementary Figure 2. Myeloid cell populations examined in the murine lungs after 4-weeks of exposure.

**a.** total CD45+ cells, **b.** viability of CD45+ cells, **c.** total monocytes and **d.** macrophages showing as % cells in the lungs of FA and PM_2.5_ exposed mice. **e-g** absolute cell counts of the macrophages in the lungs of FA and PM_2.5_ exposed mice.


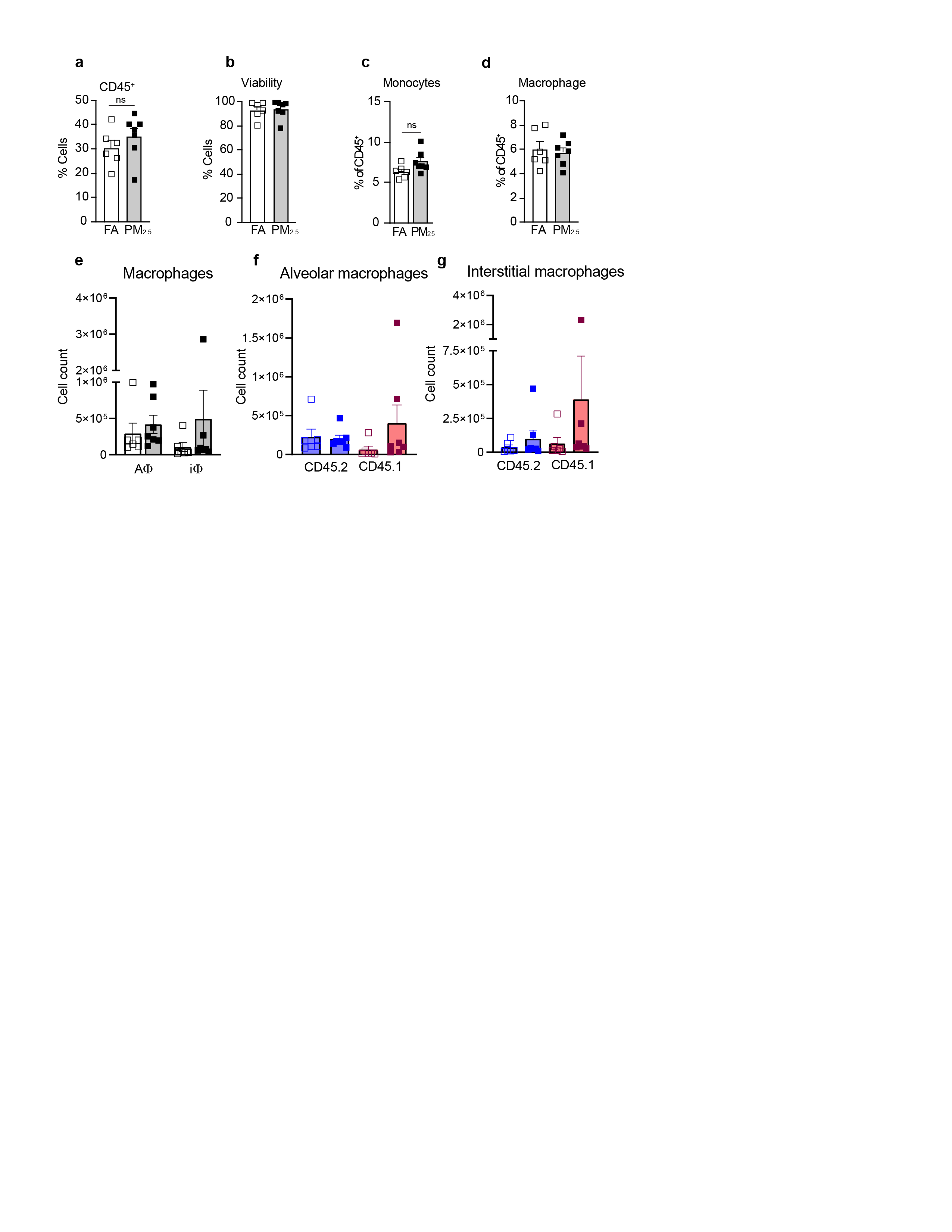


## Supplementary Figure 3. Myeloid cell populations examined in the murine lungs after 32-weeks of exposure.

**a.** total CD45+ cells, **b.** viability of CD45+ cells, **c.** total monocytes and **d.** macrophages showing as % cells in the lungs of FA and PM_2.5_ exposed mice. **e-g** absolute cell counts of the macrophages in the lungs of FA and PM_2.5_ exposed mice.

## Supplementary Figure 4. Flow cytometry gating plots.

###
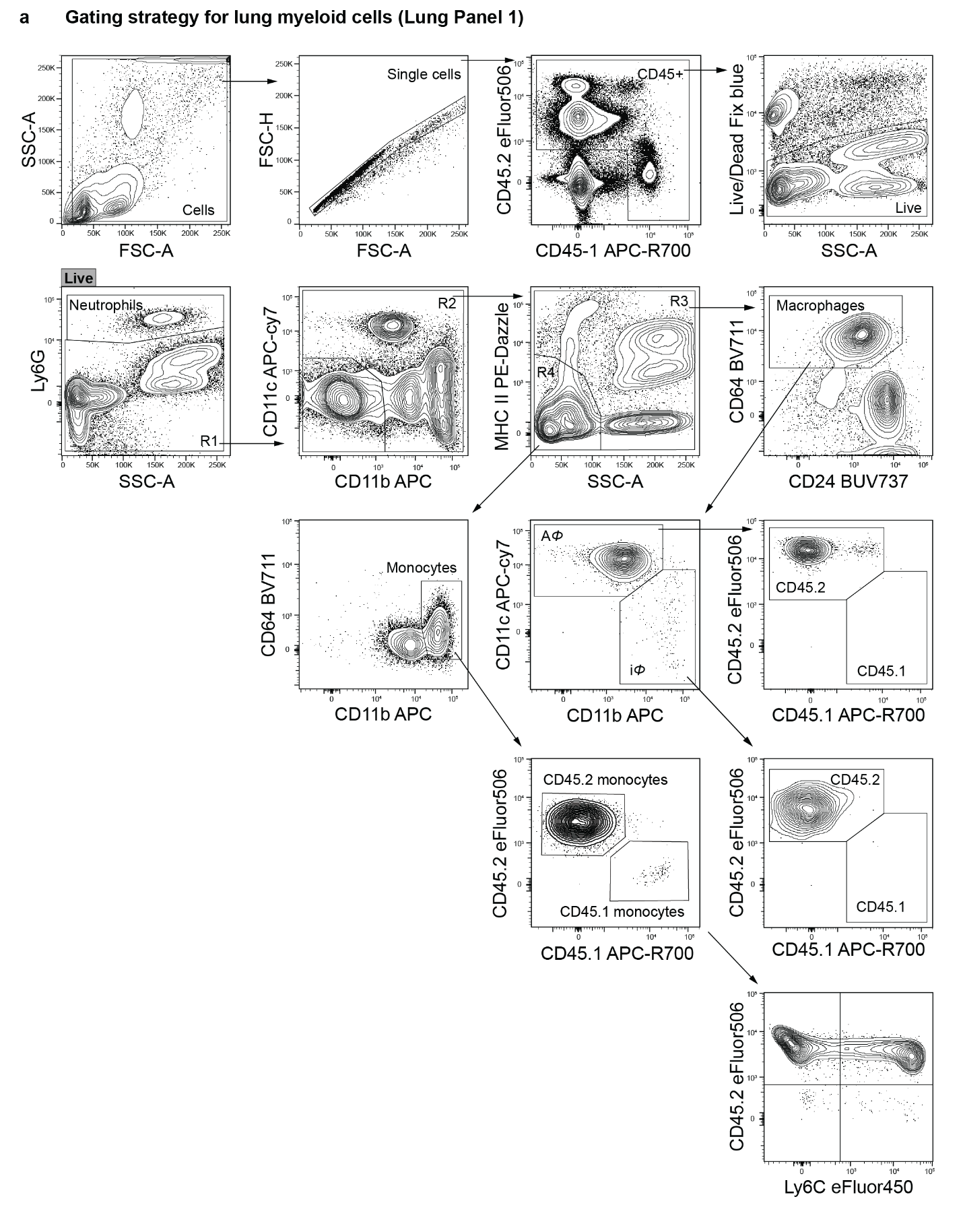
Supplementary Figure 4a. Representative flow cytometry gating strategy for mouse lung used for sorting AΦ and monocytes of CD45.1 and CD45.2 origin.


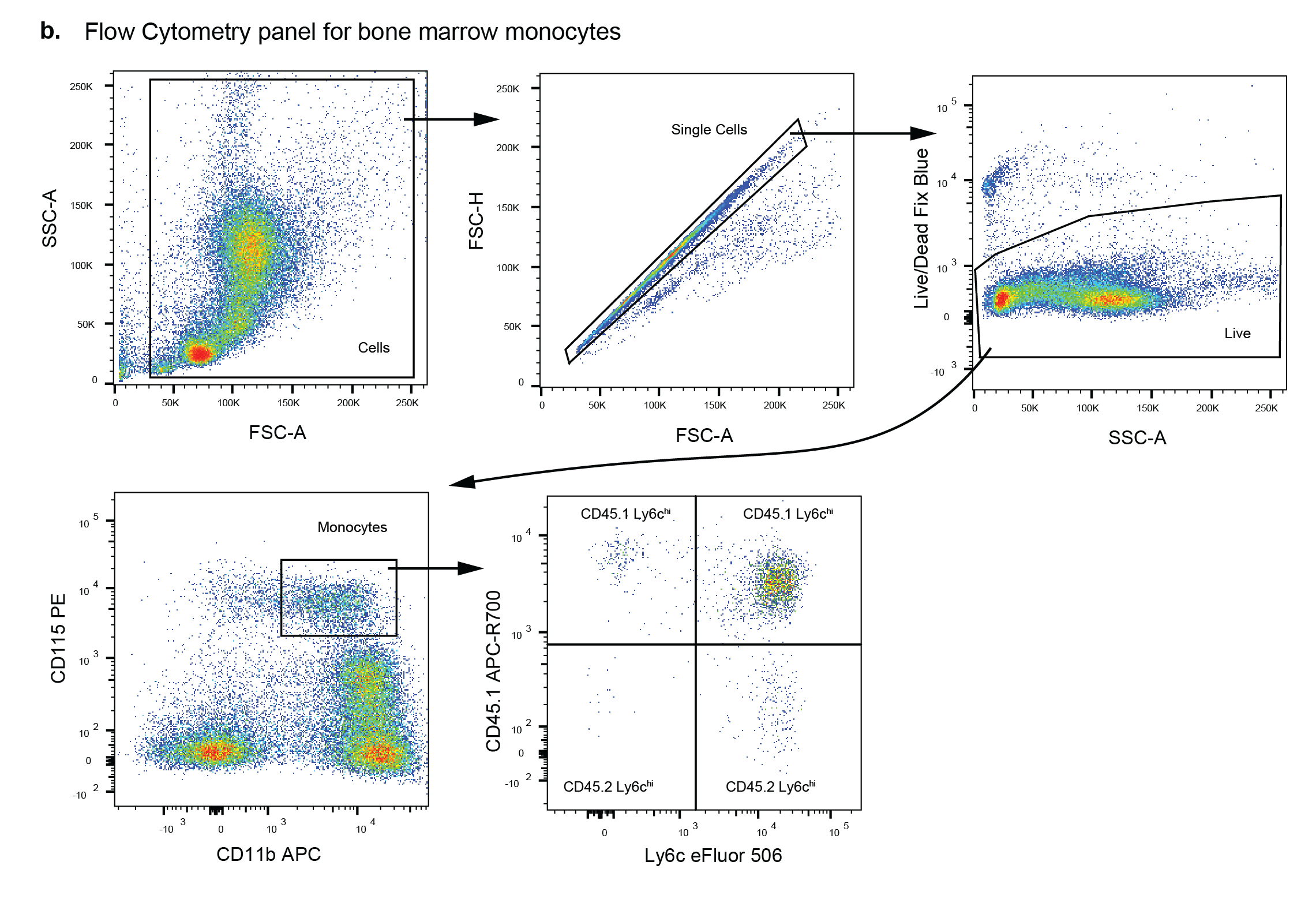


### Supplementary Figure 4b. Representative flow cytometry gating strategy for mouse bone marrow monocytes.


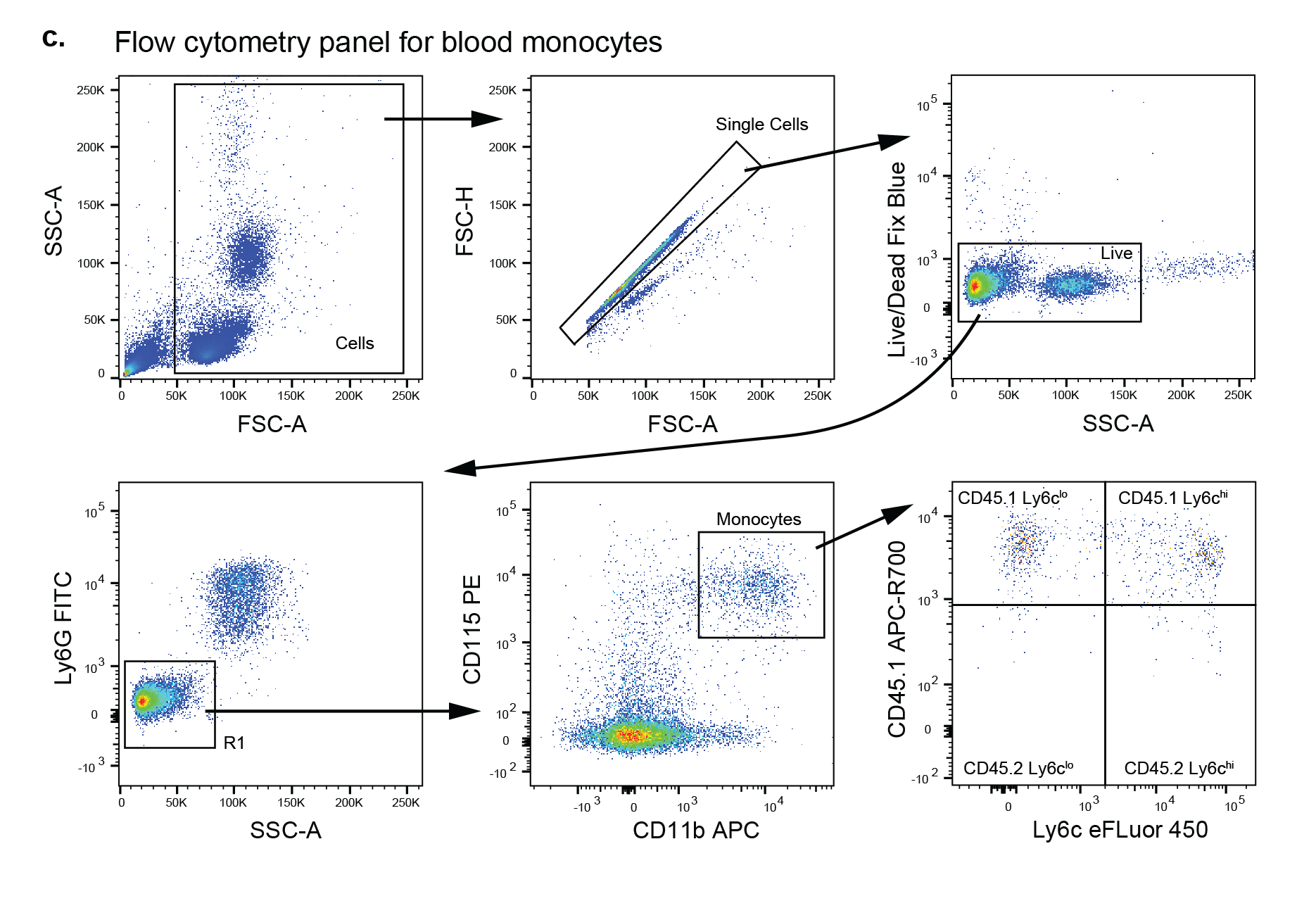


### Supplementary Figure 4c. Representative flow cytometry gating strategy for mouse blood monocytes.

**
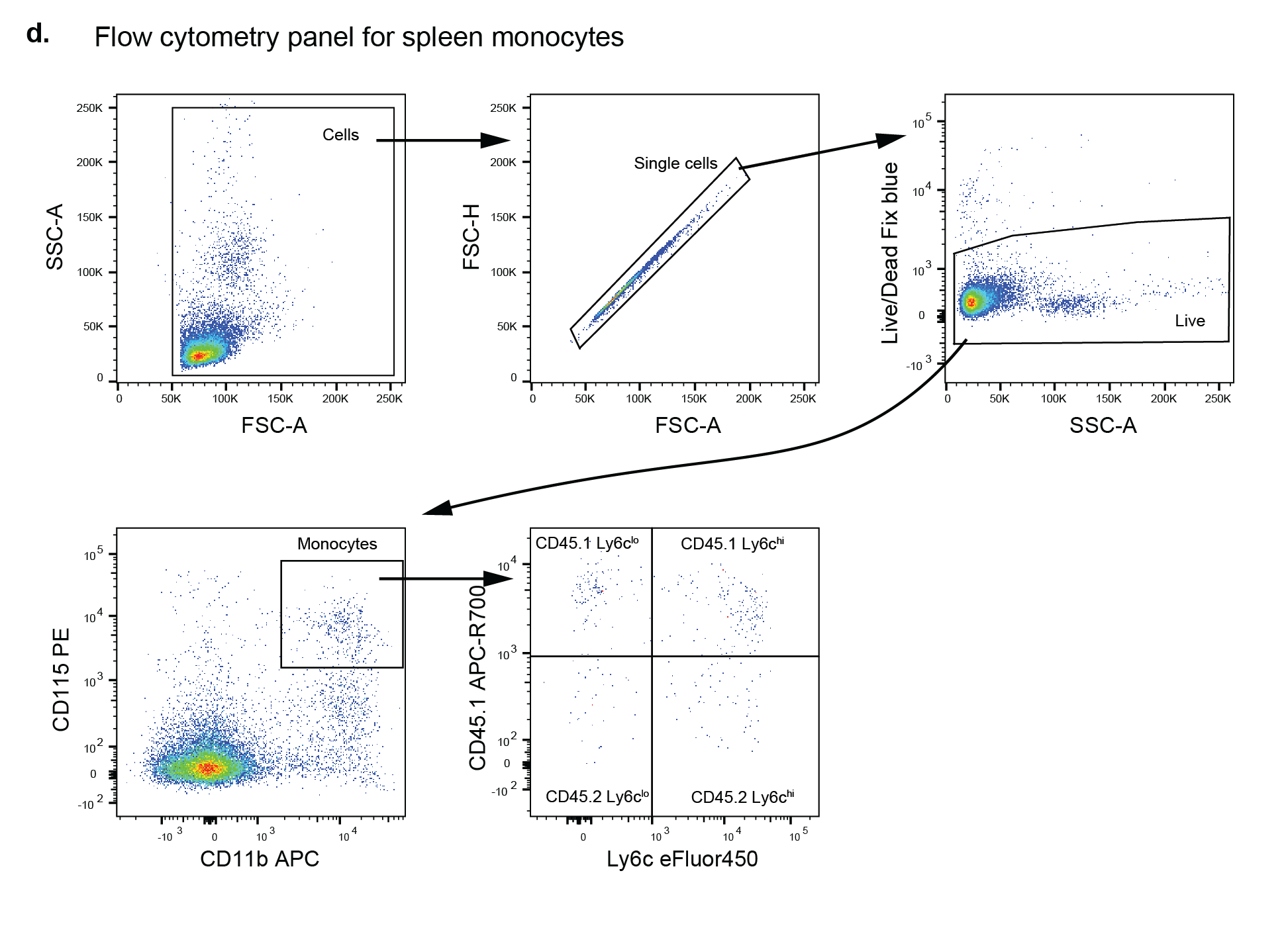
**

### Supplementary Figure 4d. Representative flow cytometry gating strategy for mouse spleen monocytes.


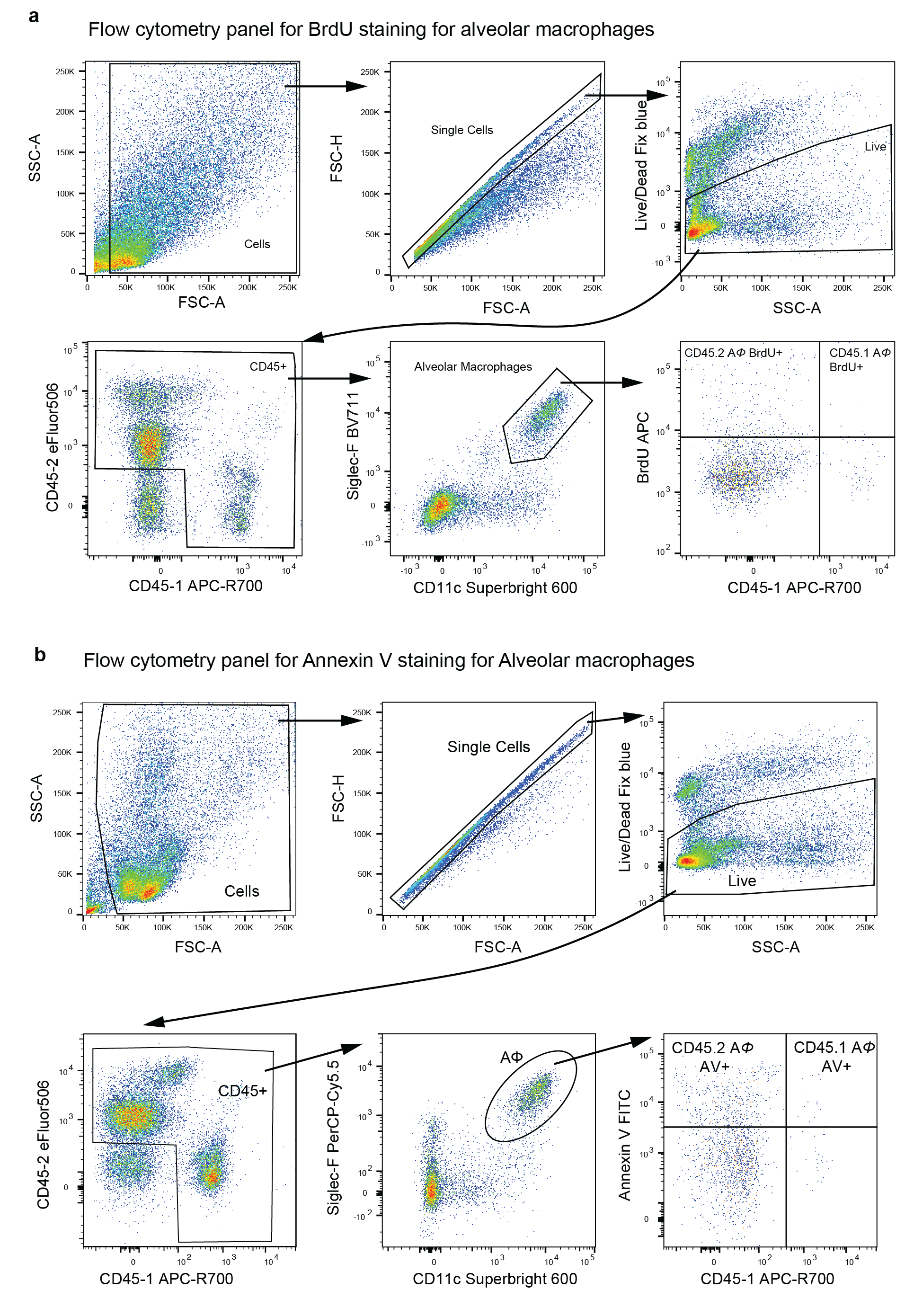


**b**


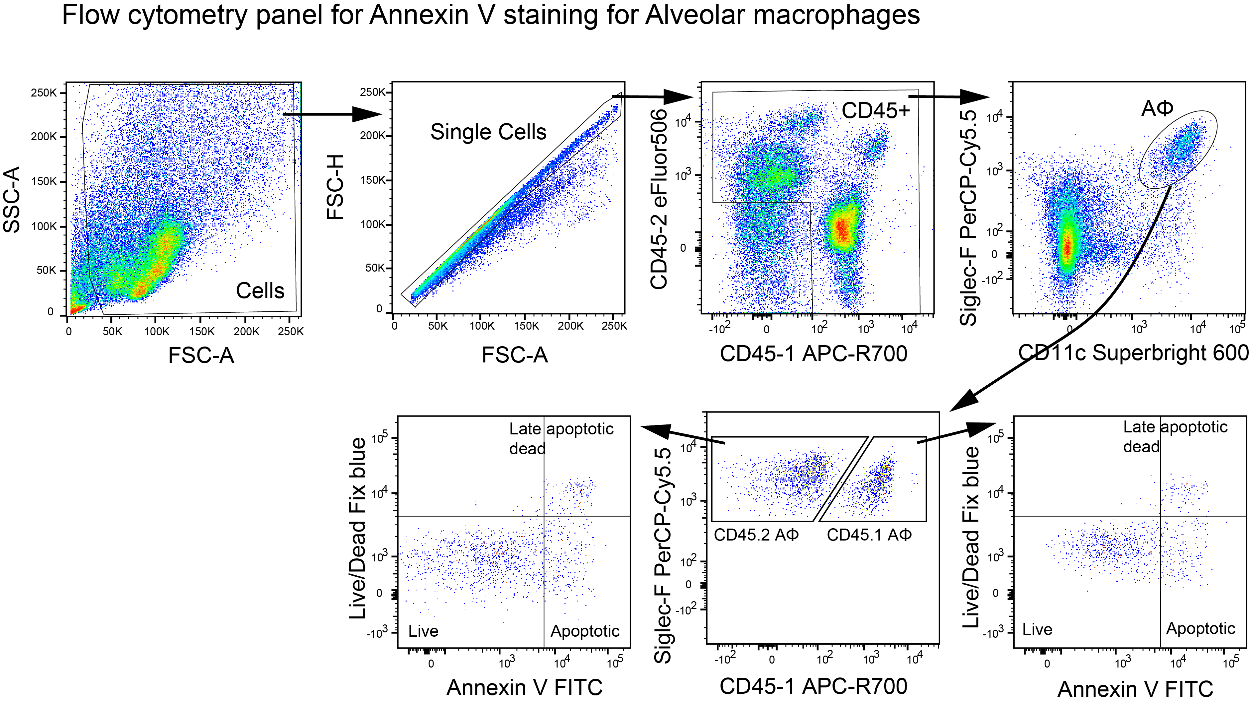


## Supplementary Figure 5. Flow cytometry panel for BrdU^+^ and Annexin V^+^ A*Φ*.

**a.** Flow cytometry panel for gating BrdU^+^ A*Φ*. **b.** Flow cytometry panel for gating Annexin V^+^ A*Φ*.

| a.  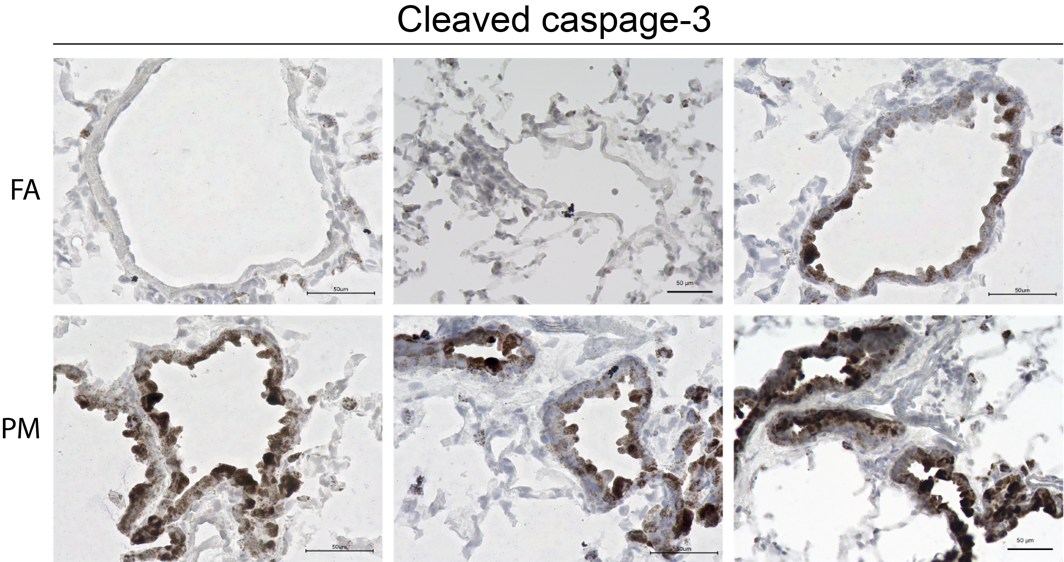 |
| --- |
| b.  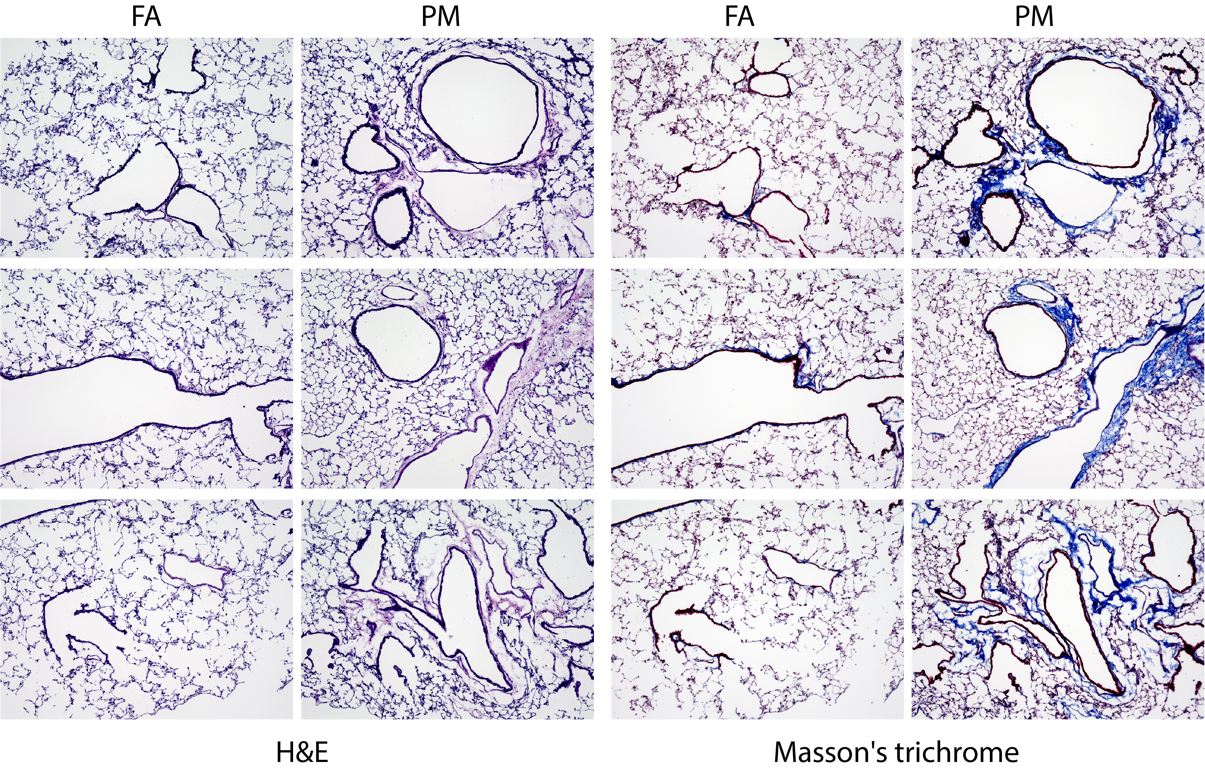 |

### Supplementary Figure 5c.

Additional representative images of lung sections stained for a. cleaved caspase-3 (n=3 mice from each group) and b. H&E and Masson’s trichrome (n=3 mice from each group).


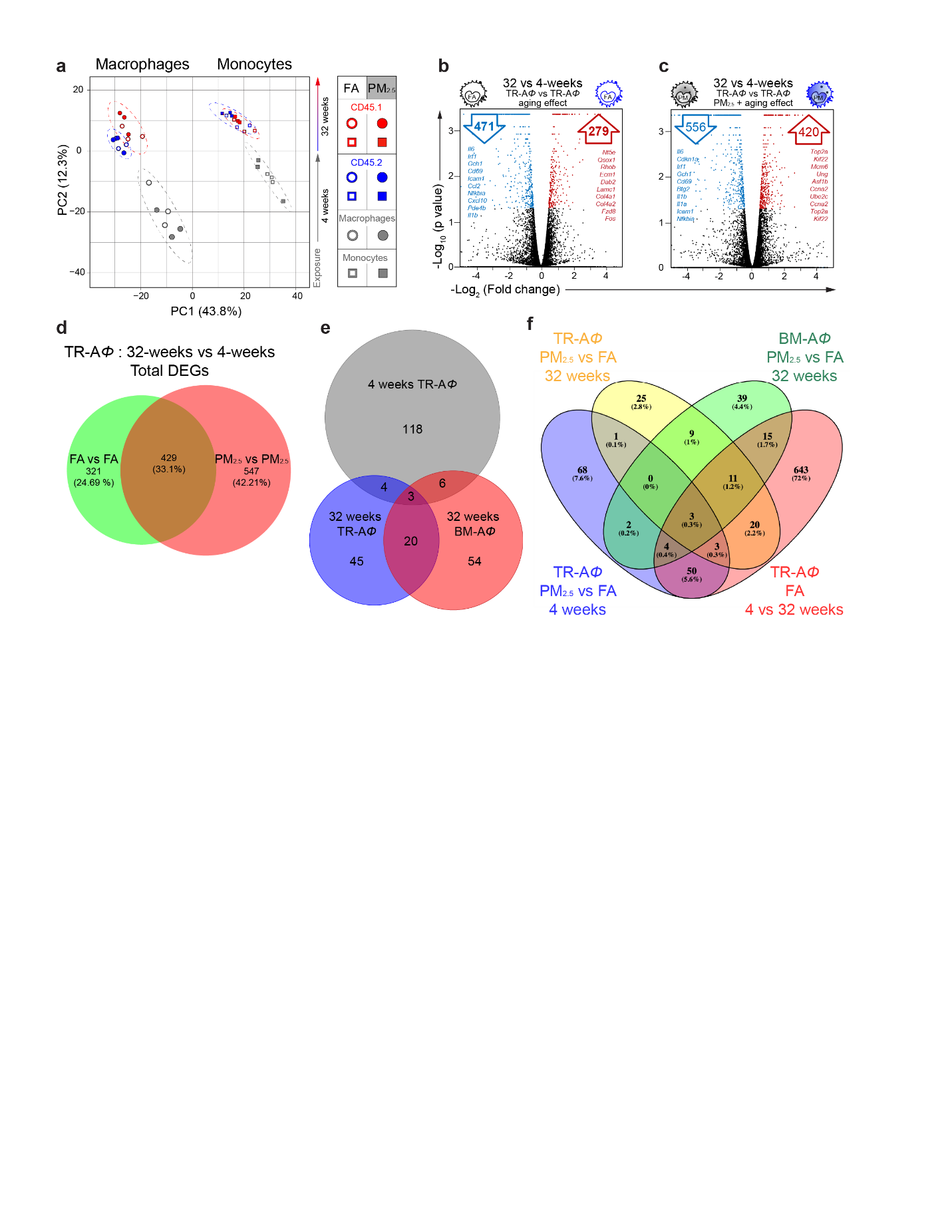


## Supplementary Figure 6. DEGs from A*Φ* of 4- and 32-weeks of FA and PM_2.5_ exposed mice.

**a.** PCA plot of flow-sorted lung monocytes and macrophages of CD45.2 and CD45.1 origin. **b.** Volcano plot of pairwise comparisons between A*Φ* of only FA exposed mice from 32 and 4-weeks. c**.** Volcano plot of pairwise comparisons between A*Φ* of only PM_2.5_ exposed mice from 32 and 4-weeks. Each volcano plot showing up- (red) and downregulated (blue) genes and top listed genes are ten Hallmark genes and/or immunological signature genes. **d.** Quantitative Venn diagram showing unique and common DEGs in 32- and 4-weeks comparisons are done in b and c. **e.** Quantitative Venn diagram showing unique and common genes from TR-A*Φ* of 4 and 32-weeks of FA/PM_2.5_ exposed mice and BM-A*Φ* of 32 weeks of FA/PM_2.5_ exposed mice. **f.** Venn diagram showing unique and common genes from different comparisons as depicted in the figure.


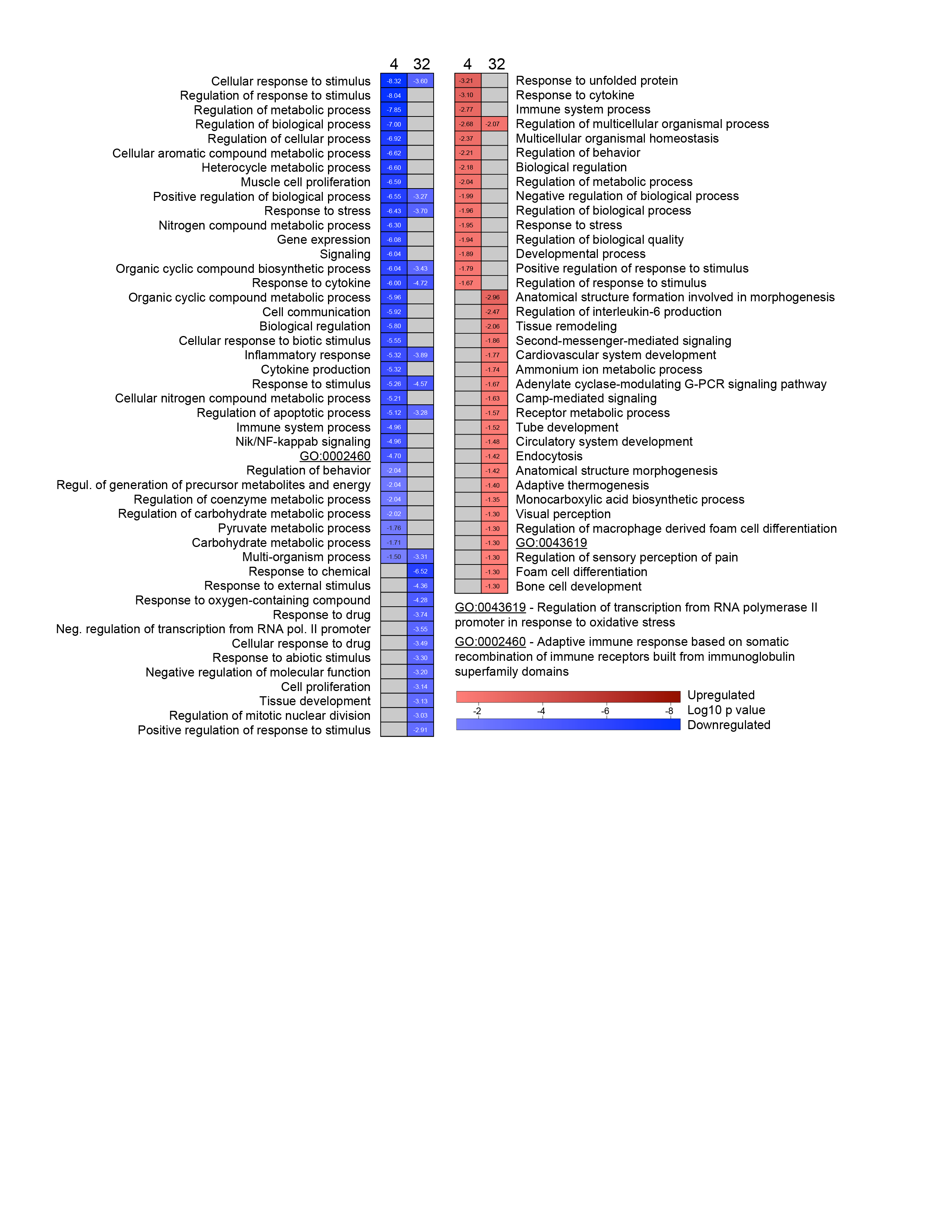


## Supplementary Figure 7. A mixed inflammatory response by A*Φ* in response to chronic PM_2.5_ exposure.

Associated GO biological process with significantly downregulated (blue) and/or upregulated (red) DEGs from A*Φ* in 4- and 32-weeks of PM_2.5_ exposure (FA vs PM_2.5_).


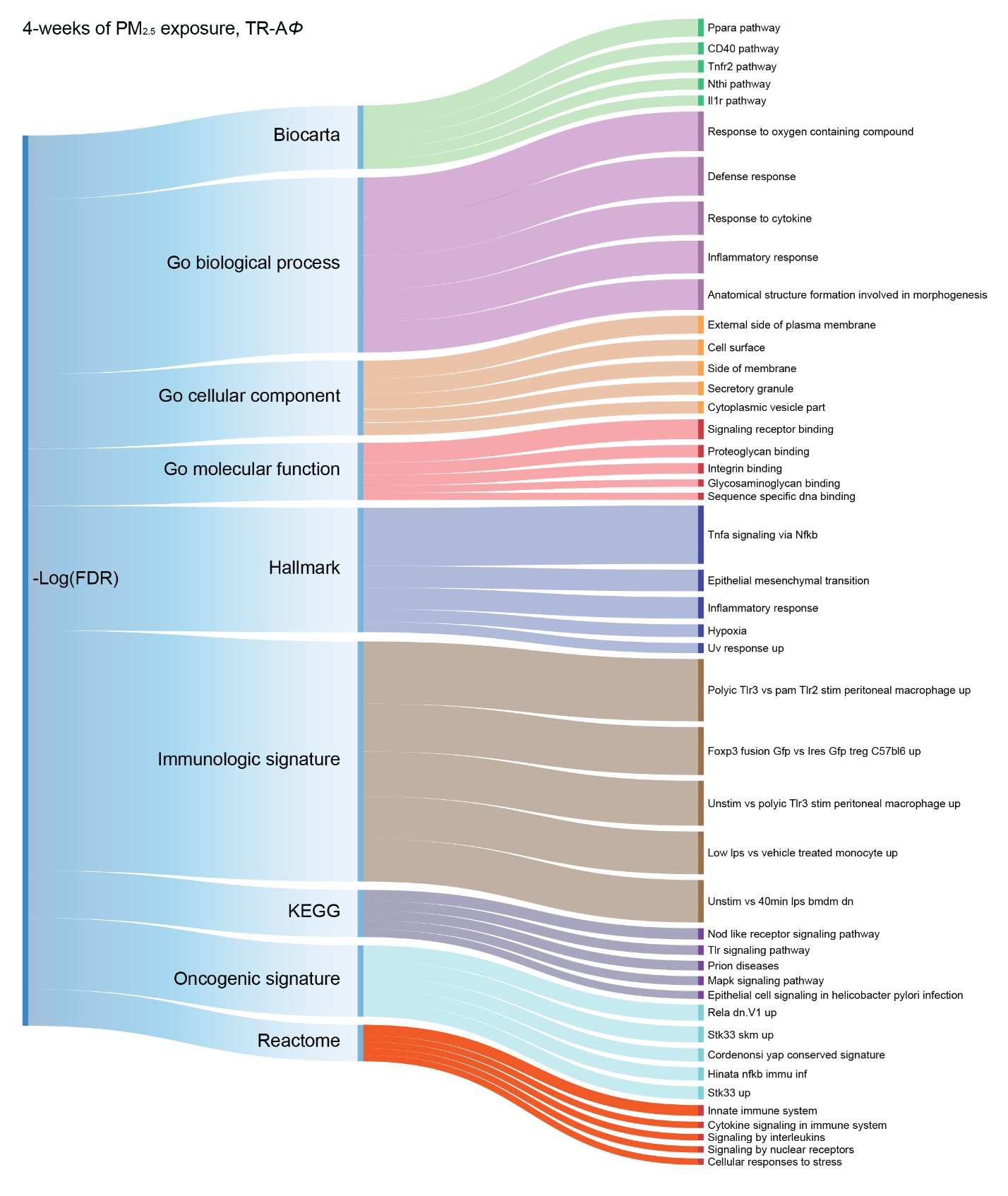


## Supplementary Figure 8a. Sanky plot of 4-weeks exposure.

Sanky plot summarizing the effect of 4-weeks of PM_2.5_ exposure on TR-A*Φ*. Top 5 significant results are listed in each category.


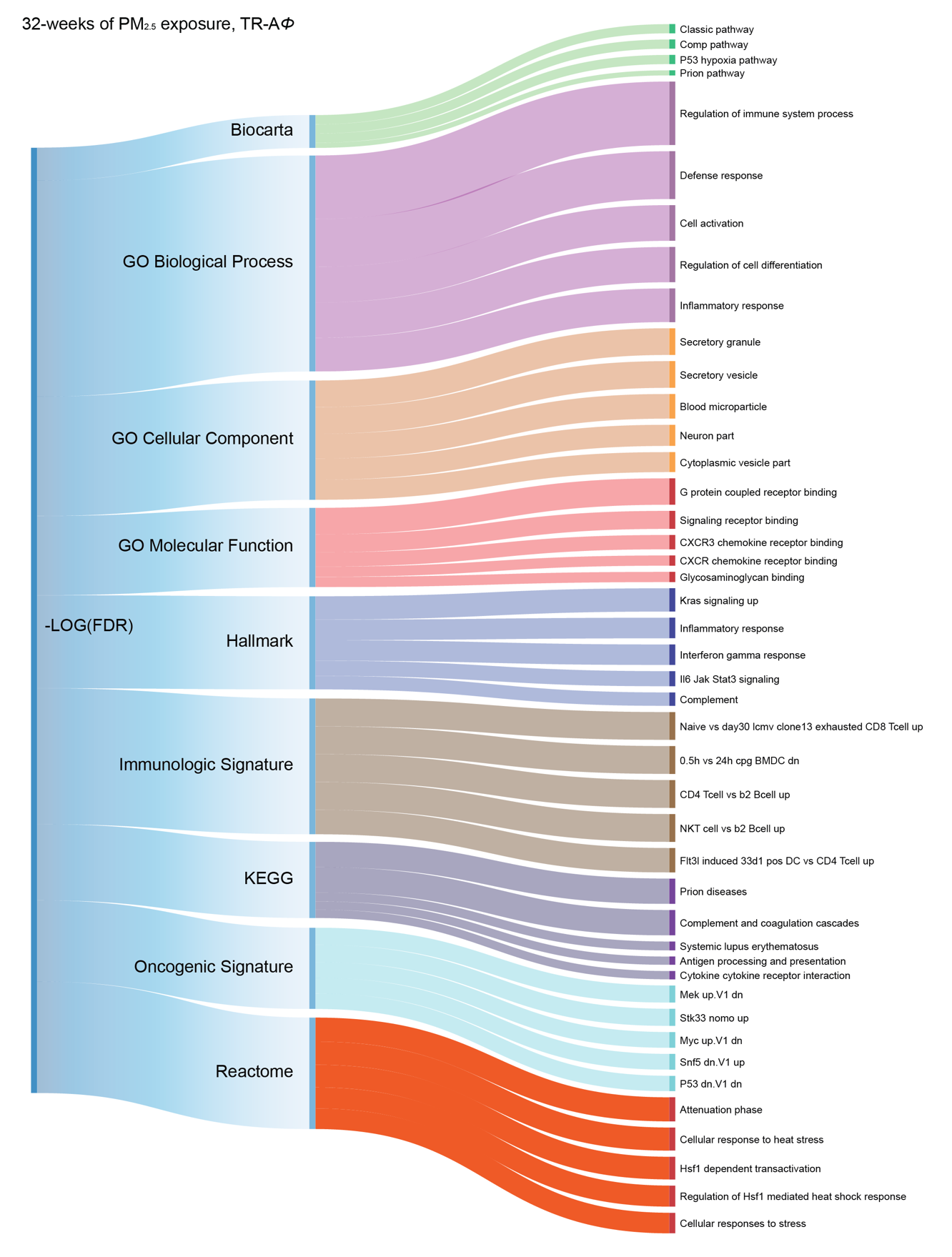


## Supplementary Figure 8b. Sanky plot of 32-weeks exposure.

Sanky plot summarizing the effect of 32-weeks of PM_2.5_ exposure on TR-A*Φ*. Top 5 significant results are listed in each category.

## Quantitative real-time RT-PCR (qPCR) analysis

To validate the RNA-seq data, total RNA was extracted from alveolar macrophages (flow sorted from 32 weeks FA/PM exposed mice) using RNeasy Micro Kit (Qiagen, Cat #74004) as per the manufacture’s instructions. cDNA was synthesized using Transcriptor First Strand cDNA synthesis kit (Roche Applied Science, Indianapolis, IN) according to the manufacturer’s protocol, using 250 ng of total RNA and random hexamers and oligo-dT primers. The amplification of few selected target genes (based on RNA-seq) was done using LightCycler® 480 SYBR Green I Master kit (Roche Applied Science, Indianapolis, IN) and gene expression was measured by quantitative real-time PCR performed on a LightCycler® 480 real-time PCR System (Roche Applied Science, Indianapolis, IN). The primers sequences were obtained from Primer bank and details of the PCR primers used in this study are shown in Supplemental Table 1. Fold changes of mRNA levels were determined using the ΔΔCt method and normalized to internal control GAPDH.


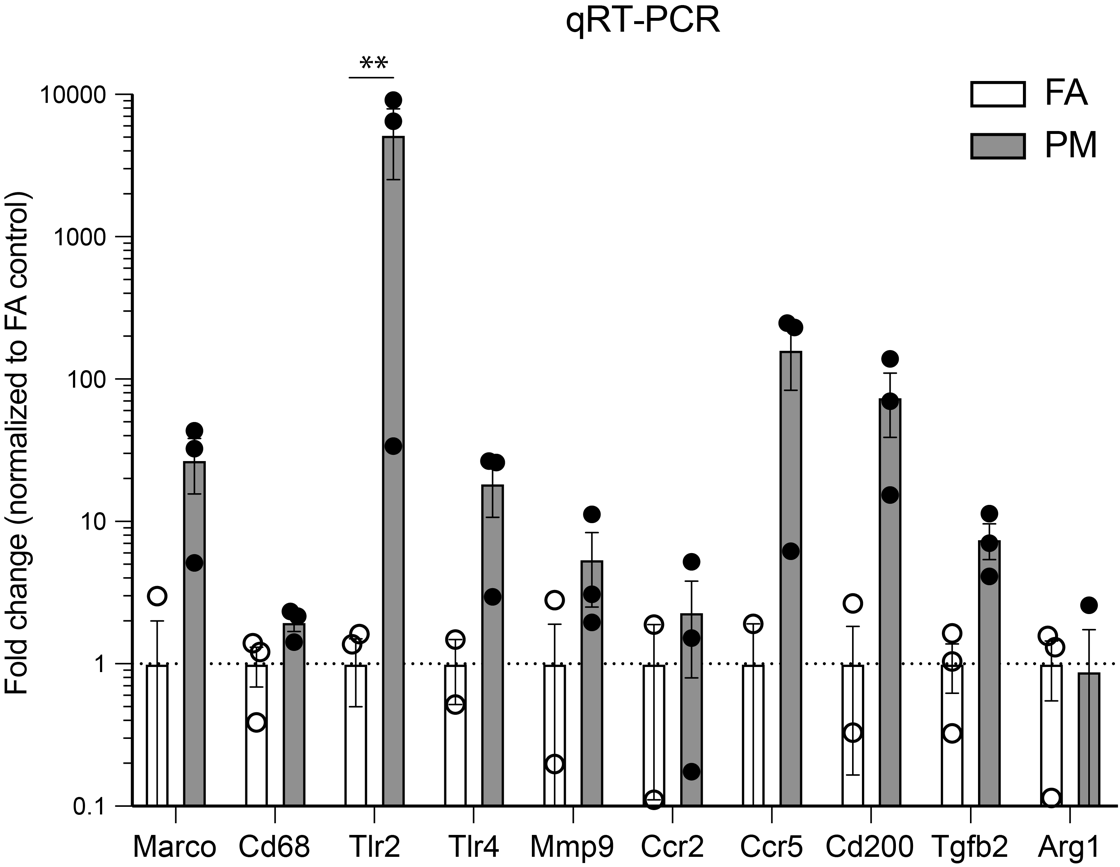


## Supplementary Figure 9. Real-Time Quantitative Reverse Transcription PCR of a few selected genes.

Expression of a few selected genes in flow-sorted alveolar macrophages of 32-weeks of PM_2.5_ exposed C57BL/6J mice. Data are represented as fold change expression over FA exposed control mean ±SEM, with from cells obtained from n=3 mice. Data were analyzed with GraphPad prism v8.3 using Student’s *‘t’* test and correct for multiple comparisons using the Holm-Sidak method. **, *p*<0.01.
